# A neuromarker for deficit syndrome in schizophrenia from a combination of structural and functional magnetic resonance imaging

**DOI:** 10.1111/cns.14297

**Published:** 2023-06-08

**Authors:** Ju Gao, Rongtao Jiang, Xiaowei Tang, Jiu Chen, Miao Yu, Chao Zhou, Xiang Wang, Hongying Zhang, Chengbing Huang, Yong Yang, Xiaobin Zhang, Zaixu Cui, Xiangrong Zhang

**Affiliations:** ^1^ Institute of Mental Health Suzhou Psychiatric Hospital, The Affiliated Guangji Hospital of Soochow University Suzhou China; ^2^ Department of Geriatric Psychiatry Nanjing Brain Hospital Affiliated to Nanjing Medical University Nanjing China; ^3^ Department of Radiology & Biomedical Imaging Yale School of Medicine New Haven Connecticut USA; ^4^ Department of Psychiatry Wutaishan Hospital of Yangzhou Yangzhou China; ^5^ Medical Psychological Institute of the Second Xiangya Hospital Changsha China; ^6^ Department of Radiology Subei People's Hospital of Jiangsu Province Yangzhou China; ^7^ Department of Psychiatry Huai'an No. 3 People's Hospital Huai'an China; ^8^ Chinese Institute for Brain Research Beijing China; ^9^ Department of Psychiatry The Affiliated Xuzhou Oriental Hospital of Xuzhou Medical University Xuzhou China

**Keywords:** classification, deficient schizophrenia, machine learning, multimodal magnetic resonance imaging, prediction

## Abstract

**Aim:**

Deficit schizophrenia (DS), defined by primary and enduring negative symptoms, has been proposed as a promising homogeneous subtype of schizophrenia. It has been demonstrated that unimodal neuroimaging characteristics of DS were different from non‐deficit schizophrenia (NDS), however, whether multimodal‐based neuroimaging features could identify deficit syndrome remains to be determined.

**Methods:**

Functional and structural multimodal magnetic resonance imaging of DS, NDS and healthy controls were scanned. Voxel‐based features of gray matter volume, fractional amplitude of low‐frequency fluctuations, and regional homogeneity were extracted. The support vector machine classification models were constructed using these features separately and jointly. The most discriminative features were defined as the first 10% of features with the greatest weights. Moreover, relevance vector regression was applied to explore the predictive values of these top‐weighted features in predicting negative symptoms.

**Results:**

The multimodal classifier achieved a higher accuracy (75.48%) compared with the single modal model in distinguishing DS from NDS. The most predictive brain regions were mainly located in the default mode and visual networks, exhibiting differences between functional and structural features. Further, the identified discriminative features significantly predicted scores of diminished expressivity factor in DS but not NDS.

**Conclusions:**

The present study demonstrated that local properties of brain regions extracted from multimodal imaging data could distinguish DS from NDS with a machine learning‐based approach and confirmed the relationship between distinctive features and the negative symptoms subdomain. These findings may improve the identification of potential neuroimaging signatures and improve the clinical assessment of the deficit syndrome.

## Graphical abstract

The present study employed multimodal neuroimaging features, alongside machine learning techniques, to investigate the classification of DS and NDS, demonstrating that the local properties of brain regions could distinguish DS from NDS. Our findings provided evidence that the classifier trained by multimodal features achieved a relatively high classification accuracy.

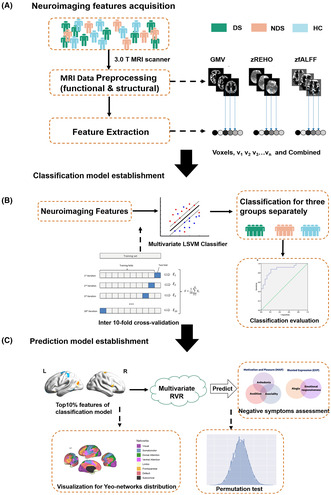

## 1 INTRODUCTION

Schizophrenia is a severe neuropsychiatric disorder with a lifetime prevalence of ~1%^1^ and constitutes a heavy global disease burden. Patients with schizophrenia experience various psychiatric symptoms causing substantial functional impairment and marked disability. A significant challenge to understanding the underlying neuropathological mechanisms of schizophrenia is the great heterogeneity in risk factors, course of illness, clinical symptoms, and treatment response.^2^, ^3^ The classification for clinical or neurobiological subtypes may be an appropriate approach to elucidating individual variabilities.

The deficit syndrome was introduced by Carpenter in 1988^4^ for distinguishing primary, enduring negative symptom, which is thought to be an integral part of schizophrenia. Increasing evidence demonstrated that deficit schizophrenia (DS) differs from non‐deficit schizophrenia (NDS) in etiopathophysiology, prevalence, biological correlations, antipsychotic treatment, and clinical outcomes.^5^, ^6^, ^7^ The Schedule for the Deficit Syndrome (SDS)^8^ is frequently used to classify patients into those with and without deficit syndrome. However, a significant limitation of the SDS for evaluating deficit syndrome is that clinicians need the information based on a cross‐sectional and longitudinal assessment of negative symptoms and should be familiar with the course of illness in 12 months, which is a significant barrier to identifying deficit syndrome in early stages of the disease.

Structural and functional magnetic resonance imaging (sMRI and fMRI) have been widely used to study schizophrenia. Evidence from previous studies showed that compared to NDS, DS exhibited a more widespread cortical thinning pattern in the left temporoparietal junction^9^, ^10^ and distinct degrees of cortical gyrification in the left inferior parietal lobule.^11^ Based on graph theory network analysis, higher nodal connectivity in the right inferior temporal gyrus in DS was also reported.^12^ These evidences provide insights into the heterogeneity of schizophrenia subtypes, suggesting that the deficit syndrome is inherent in unique neural mechanisms more than phenomenological characteristics. Despite increased attention, current MRI investigations of DS have often focused on a single neuroimaging modality and the results remained largely inconsistent. Leveraging the cross‐information encoded in multimodal MRI data to investigate the deficit subtypes would improve the identification of potential neuroimaging signatures and compensate for the limitations of clinical assessments.

Recently, neuroimaging measurements coupled with machine learning techniques have been successfully used to identify patients and predict continuous clinical traits of schizophrenia.^13^, ^14^, ^15^ Using multivariate analysis based on the gray matter volume, the patients of schizophrenia reported a discriminative accuracy of 76%, and multiple disease‐related neuroimaging signatures including the medial prefrontal, temporal lobe, and lateral cortex were revealed.^13^ Moreover, recent studies furtherly identified biologically plausible biotypes within schizophrenia.^16^ For instance, Chen and colleagues built a multivariate classification model and achieved an out‐of‐sample classification accuracy of 70% for two core psychopathological subtypes of schizophrenia.^17^ These literature provided important insight for classifying the deficit subtype of schizophrenia and further identifying brain‐based neuroimaging biomarkers.

In our study, we included individual structural and functional measures including GMV, fALFF, and REHO as features for machine learning classification, due to their well‐established relevance to the research question and their capability to capture different aspects of brain morphological and functional activity characteristics. The fALFF reflects the amplitude of low‐frequency fluctuations in the resting‐state fMRI signal and has been extensively utilized in investigating functional abnormalities in various psychiatric disorders.^18^, ^19^, ^20^ It provides valuable insights into spontaneous brain activity, indicating potential alterations in neural synchronization and connectivity. On the contrary, REHO measures the regional similarity or homogeneity of the fMRI time series within specific brain regions. It is sensitive to local temporal coherence and can identify areas with abnormal neural synchronization patterns, making it particularly useful in capturing local functional abnormalities in different brain disorders.^18^, ^21^, ^22^ By incorporating these features, along with brain structural information, into machine learning classification model, a more comprehensive characterization of the underlying neural mechanisms associated with the deficit schizophrenia population can be achieved.

**FIGURE 1:**
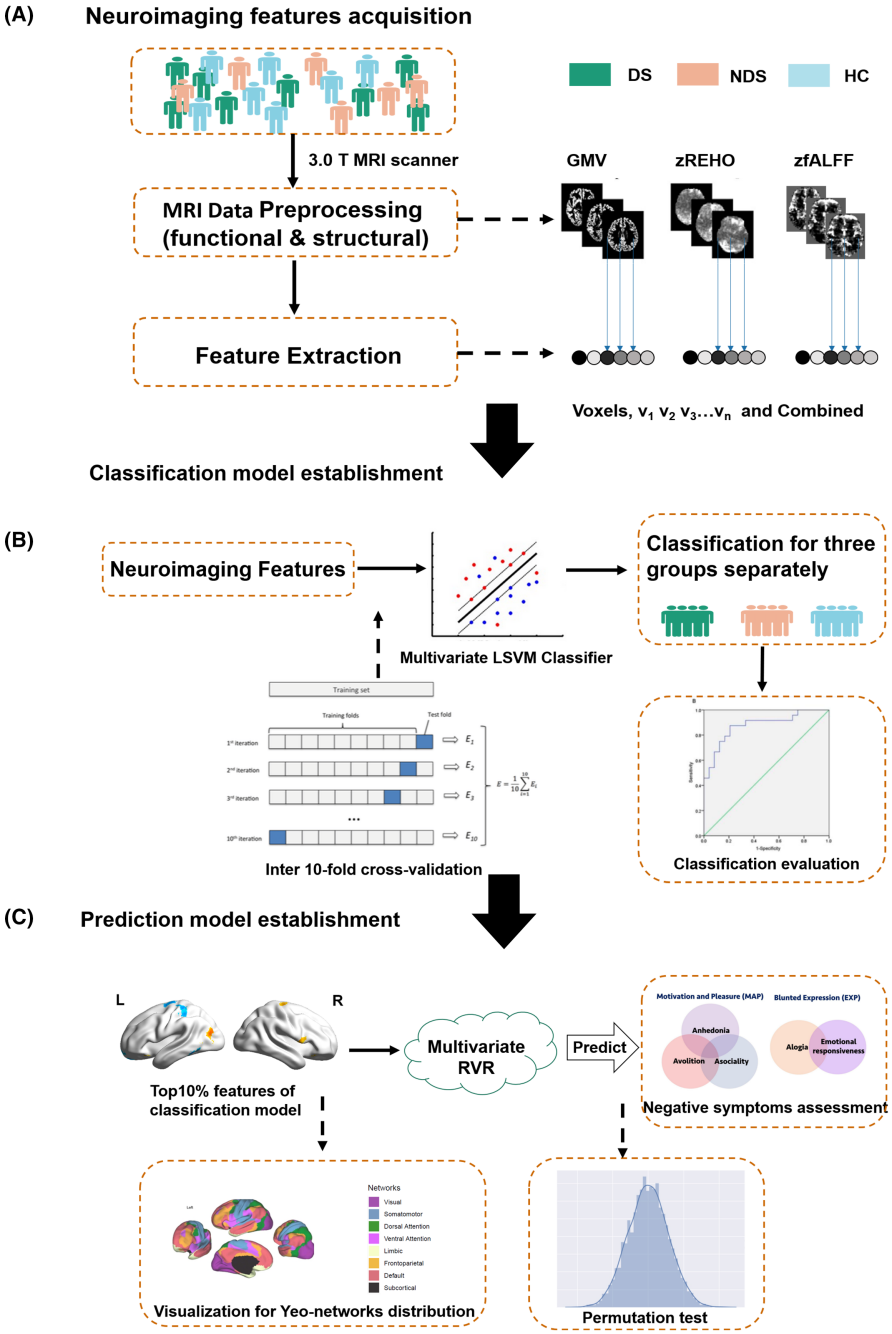
Schematic overview of the data analysis pipeline. (A) Imaging data preprocessing and feature extraction with each voxel based on GMV, zfALFF, and zREHO maps. (B) Overview of the classification framework in DS, NDS, and HC groups. (C) Establishment of the prediction model using the distinctive feature of the classification model of DS and NDS groups.

A schematic overview of the study design and analysis pipeline is shown in Figure 1. Considering the possible influence of gender differences and hormone levels,^23^ the present study only recruited male patients with schizophrenia.

## 2 MATERIALS AND METHODS

### 2.1 Participant information

A total of 183 patients with schizophrenia were recruited from the Psychiatric Rehabilitation Unit of Yangzhou Wutaishan Hospital, Jiangsu Province, China. All subjects were male, right‐handed Han Chinese. The patients were diagnosed with schizophrenia according to the Diagnostic and Statistical Manual of Mental Disorders, Fourth Edition (DSM‐IV). The Chinese version of the Schedule for the Deficit Syndrome (SDS)^24^ was used to diagnose DS. The patients with NDS were regarded as those not conforming to SDS criteria. Healthy controls (HC) were recruited from the community and matched for age, gender, and educational level. The researcher explained the purpose and significance of this study to all subjects and their legal guardians, and after obtaining consent, the subjects themselves or their legal guardians signed an informed consent form. This study was approved by the Ethics Committee of Nanjing Brain Hospital and the Ethics Committee of Yangzhou Wutaishan Hospital.

### 2.2 Clinical measurements

The Brief Psychiatric Rating Scale (BPRS), organized into positive, negative, disorganized, and affect syndromes based on the findings of the factor analysis of 18‐item,^25^ was used to evaluate the whole spectrum of symptoms in schizophrenic patients. For a comprehensive reflection of the negative symptoms, the Scale for the Assessment of Negative Symptoms (SANS) was used to assess the negative symptoms.^26^, ^27^ The SANS consists of 19 items, representing five rationally derived domains: Affective Flattening or Blunting, Alogia, Avolition‐Apathy, Anhedonia‐Asociality, and Inattention. Negative symptoms of SANS are divided into motivation and pleasure (MAP) and diminished expressivity (EXP) subdomains (Appendix S1). Inattentiveness items (Work inattentiveness and Inattentiveness during mental status testing) were the least correlated with other items in SANS‐19 (Figure S1), so in the data analysis of the scale, we mainly studied the two subdomains of MAP and EXP. The positive symptoms were evaluated using the Scale for the Assessment of Positive Symptoms (SAPS).

### 2.3 MRI data acquisition

The MRI scans were obtained using a 3.0 T MRI scanner (GE HDx) with an 8‐channel phased‐array head coil in the Subei Hospital of Jiangsu Province, Yangzhou, China. Both sMRI and sMRI data were collected. During MRI scanning, the subjects were asked to lie quietly with eyes‐closed wakefulness. The details of scanner parameters are described in Appendix S1.

### 2.4 Imaging processing pipeline

The resting‐state fMRI data were preprocessed using the Statistical Parametric Mapping 12 package (http://www.fil.ion.ucl.ac.uk/spm/spm12) and the toolkit of Data Processing and Analysis for Brain Imaging (DPABI).^28^ Considering the magnetization equilibrium, the first 10 volumes were excluded. Slice timing was performed to correct the acquisition time delay among the remaining 230 volumes, and then the volumes were realigned to the first volume for head motion correction. The criterion for the head motion was set as 3 mm in translation and 3° in rotation. Native T1 images were segmented and coregistered to the resting‐state functional images and then spatially normalized to the standard space of the Montreal Neurological Institute (MNI) using an optimum 12‐parameter affine transformation and nonlinear deformations. Subsequently, the functional images were normalized to MNI space according to the transformation parameters obtained from structural images and further resampled to an isolated resolution of 3 × 3 × 3‐mm cubic voxel. A linear trend of the time courses was removed from each voxel, and several nuisance signals (involving Friston's 24‐head motion parameters, cerebrospinal fluid signal, white matter signal, and global mean signal) were regressed out.

After data preprocessing, three local properties of brain regions extracted from sMRI and fMRI imaging were calculated as model features. (1) Gray matter volume (GMV). GMV provides the structural information of brain and was calculated through the CAT12 toolbox (http://dbm.neuro.uni‐jena.de/cat12) running within SPM12 (http://www.fil.ion.ucl.ac.uk/spm). (2) Fractional amplitude of low‐frequency fluctuations (fALFF). Since the low‐frequency fluctuations across 0.01–0.08 Hz are of particular relevance to the resting‐state fMRI, the fALFF was calculated to examine regional spontaneous brain activity.^29^ (3) Regional homogeneity (REHO). Individual REHO maps were calculated by computing Kendall's concordance coefficient (KCC), which measures the BOLD time series for each voxel and the nearest 26 contiguous voxels, helping reveal the complexity of brain function.^30^ fALFF and REHO were calculated using DPABI.^28^ The details of the calculation are shown in Appendix S1.

### 2.5 Classification models establish

We applied a multivariate pattern analysis to distinguish DS from NDS, as well as distinguish schizophrenia patients from HCs. To quantify the classification accuracy, a stratified 10‐fold cross‐validations (10 F‐CV) framework was used to evaluate the classifiers (https://github.com/ZaixuCui/Pattern_Classification/blob/master/SVC/SVM_2group_10Folder.m). In brief, the full data was divided into 10 folds, where 9 folds were used for training, and one was used for testing. To mitigate the influence of data divisions, we performed the 10 F‐CV analysis 101 times for each model and reported the median as the final result. The performance of each classifier was assessed in terms of accuracy, sensitivity, specificity, positive decision value and negative decision value. We carried out 1000‐time permutation tests to examine the significance of prediction performance. The most discriminative features were defined as the top 10% of features gaining the greatest weight.

### 2.6 Prediction model construction

Multivariate relevance vector regression (RVR) with a linear kernel was used to explore the neural mechanisms underlying the characteristics of negative symptoms in DS and NDS. The top 10% discriminative features in the classification model were concatenated to yield a new input feature vector for the RVR model, and leave‐one‐out cross‐validation (LOOCV) was employed to estimate the generalizability. *N*‐1 subjects were used as the training set, and the left one was used as the testing set. Each feature was scaled to a range of zero to one across the training set, and the feature vector of the testing subject was scaled using the same scaling parameter^31^ (https://github.com/ZaixuCui/Pattern_Regression_Matlab/tree/master/RVR). By applying the predictive model constructed in the training set to the testing subset, we obtained the predicted score for the testing subject. The training and testing procedures were repeated *N* times so that each subject was used once as the testing subject. Subsequently, 1000‐times permutation tests that shuffle the correspondence between behavioral scores across the neuroimaging features were performed to confirm the final *r* and mean absolute error (MAE) was significant compared to by chance.

### 2.7 Statistical analysis

Statistical analysis was performed using SPSS version 19.0 (SPSS Inc., USA). We performed Kolmogorov–Smirnov test to assess whether data follows a normal distribution. The differences in continuous variables, such as demographic and clinical characteristics and neurocognitive assessment between DS, NDS, and HCs were determined through one‐way analysis of variance (ANOVA) and post hoc pairwise comparisons. Psychiatric symptoms were compared between DS and NDS groups using two‐sample t‐tests.

A larger absolute value of the weight of the corresponding feature indicated a greater contribution to classification in the context of all other features. Therefore, the feature was selected if the absolute value of its weight was in the top 10%. This threshold could eliminate noise components to some extent, enabling better identification of the most discriminative regions. Finally, we set a cluster size of 2400 mm^3^ to better visualize the most discriminative features for each metric.

## 3 RESULTS

### 3.1 Demographics and clinical data

Thirty subjects (including 16 DS, 12 NDS, and 2 HC) were excluded because translation and rotation exceeded ±3 mm or 3° or missing layers of image. There were no significant differences in age, gender, and head motion among the three groups (*p* > 0.05). The education level of DS was significantly lower than that in the controls, whereas no significant difference was found between the two schizophrenia subgroups. No statistically significant differences were observed in age, age at onset, and CPZ‐equivalent dose between DS and NDS. DS showed more severe syndrome in negative symptoms relative to NDS, but not in positive, disorganization and affect syndrome in BPRS (Table 1). Significant differences in each item except work inattentiveness and inattentiveness were found between DS and NDS patients (Table S1).

**TABLE 1:** Demographics and clinical data for DS, NDS, and HC groups.

	DS (*n* = 66)	NDS (*n* = 89)	HC (*n* = 118)	*F*/*t*	ANOVA/*t*‐tests *p* value	Post hoc *p‐*value (DS vs. NDS)
Age	51.45 ± 8.16	49.18 ± 7.96	50.76 ± 7.60	1.794	0.168	0.227
Education years	8.45 ± 3.01^△^	8.91 ± 2.47	9.76 ± 2.82	5.322	0.005	0.993
Age at onset	22.50 ± 3.51	23.08 ± 4.48	–	−0.869	0.386	
Duration	28.65 ± 7.74^#^	26.10 ± 8.01	–	1.988	0.049	
CPZ‐equivalent dose (mg/day)	501.21 ± 235.49	570.17 ± 208.48	–	−1.926	0.056	
BPRS total score	31.33 ± 3.22*	27.26 ± 3.13	–	7.908	<0.001	
Positive syndrome	6.26 ± 1.13	6.33 ± 1.36	–	−0.333	0.74	
Negative syndrome	11.82 ± 2.42*	7.63 ± 1.35	–	12.684	<0.001	
Disorganized syndrome	6.45 ± 1.18	6.30 ± 1.08	–	0.828	0.409	
Affect	6.80 ± 1.15	7.00 ± 1.32	–	−0.967	0.335	
SAPS total score	8.91 ± 3.64	9.72 ± 4.54	–	−1.193	0.235	
SANS total score	55.64 ± 11.64*	33.89 ± 8.80	–	12.719	<0.001	
Jenkinson head motion	0.12 ± 0.09	0.10 ± 0.07	0.09 ± 0.07	2.813	0.062	0.809
BMI	24.25 ± 3.53	24.78 ± 3.19	24.03 ± 2.24	1.716	0.182	0.794

*Note*: Values represented as mean ± SD.

Abbreviations: BMI, Body Mass Index; BPRS, Brief Psychiatric Rating Scale; CPZ, chlorpromazine; DS, deficit schizophrenia; HC, healthy controls; NDS, non‐deficit schizophrenia; SANS, the Scale for the Assessment of Negative Symptoms; SAPS, the Scale for the Assessment of Positive Symptoms; SDS, the Schedule for the Deficit Syndrome.

*p < 0.001 DS vs. NDS; ^△^ *p* < 0.05 DS/NDS vs. HC; ^#^ *p* < 0.05 DS vs. NDS.

### 3.2 Multivariate pattern support vector classification analyses

**FIGURE 2:**
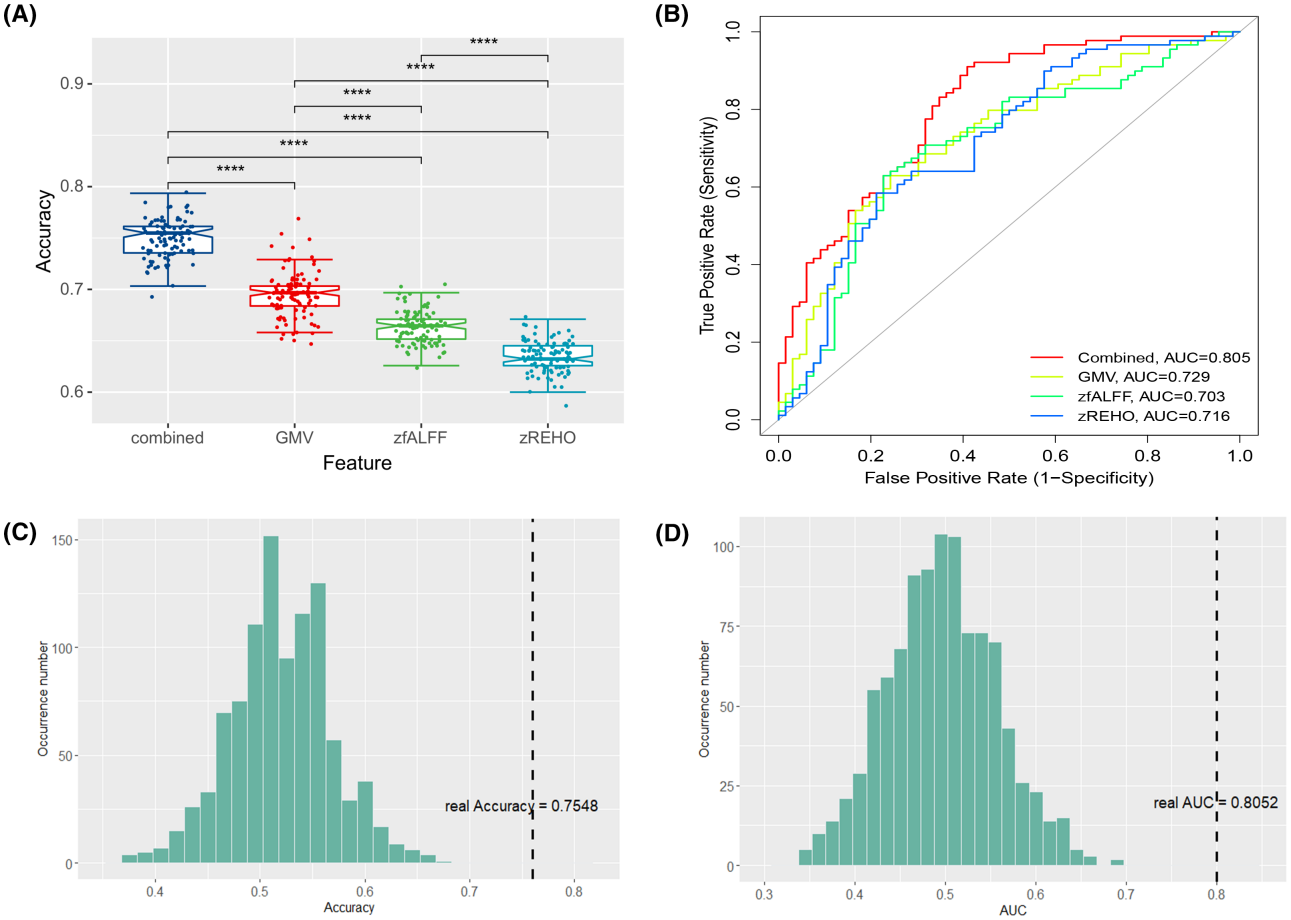
Linear SVM analysis results. (A) Comparison of the accuracy of SVM classification between DS and NDS groups (101 times 10‐fold cross‐validation results). (B) Receiver operating characteristics curves for classification performance of classifiers trained on combined and single modality features. (C, D) Permutation test results showed the distribution of actual accuracy and AUC of the SVM combined classifier between DS and NDS groups.

To determine which classifier better distinguishes DS from NDS, single‐modal and multimodal features were evaluated by the SVM methods. The combined classifier achieved higher accuracy (75.48%) and AUC (0.81) compared with the single model (Table 2). The 101 times 10 F‐CV results of the combined model exhibited significantly higher accuracy and AUC than the single modality model (Figure 2A,B). The permutation tests revealed that the accuracy and AUC (*p*_accurancy_ <0.001, *p*_AUC_ <0.001) were significant compared to by chance (Figure 2C,D).

**TABLE 2:** Results of classification models using the combined feature and single feature.

	Accuracy (%)	Sensitivity (%)	Specificity (%)	PPV (%)	NPV (%)	AUC
DS vs. NDS						
Combined	75.48	60.61	86.52	76.92	74.76	0.81
GMV	69.68	59.09	77.53	66.10	71.88	0.73
zfALFF	66.45	54.55	75.28	62.07	69.07	0.70
zREHO	63.87	50.00	74.16	58.93	66.67	0.72
DS vs. HC						
Combined	94.02	87.88	97.46	95.08	93.50	0.99
GMV	86.41	78.79	90.68	82.54	88.43	0.90
zfALFF	93.48	90.91	94.92	90.91	94.92	0.99
zREHO	92.93	89.39	94.92	90.77	94.12	0.99
NDS vs. HC						
Combined	89.86	82.02	95.76	93.42	87.60	0.96
GMV	84.06	79.78	87.29	82.56	85.12	0.91
zfALFF	85.51	82.02	88.14	83.91	86.67	0.93
zREHO	88.41	83.15	92.37	89.16	87.90	0.94

Abbreviations: AUC, area under the curve; NPV, negative predictive value; PPV, positive predictive value.

The same analyses were performed to distinguish schizophrenia subgroups from the HCs. The results showed that all the models achieved significant accuracies (ACC = 84.0–93.48%, AUC = 0.90–0.99), and the multimodal models generally outperformed the single‐modality models (Table 2, Figures S2 and S3).

### 3.3 Contributing features and corresponding weights

**FIGURE 3:**
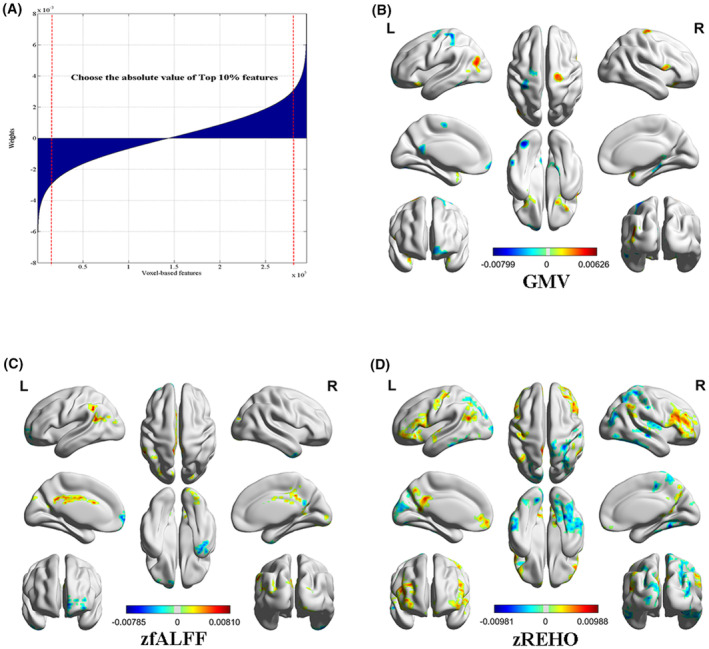
(A) Selection of the top 10% absolute value of its weight across all features. Discriminative weights of voxel‐wise features in (B) gray matter volume (GMV), (C) *z*‐normalized fractional amplitude of low‐frequency fluctuations (zfALFF), (D) *z*‐normalized regional homogeneity (zREHO).

The brain regions that contributed most to the classification between DS and NDS were identified by extracting the top 10% weighted features in multimodal models. Totally, there were 36 discriminative features identified, including 11 GMV, 8 zfALFF, and 17 zREHO clusters (Figure 3 and Table S2). The GMV pattern primarily included the following region: bilateral inferior frontal gyrus orbital part, bilateral superior temporal gyrus, bilateral precentral gyrus, left fusiform gyrus, right parahippocampal gyrus, left inferior temporal gyrus, left superior frontal gyrus, left middle occipital gyrus, left middle temporal gyrus, right inferior frontal gyrus opercular part, left postcentral gyrus, and cerebellum lobule IV/V. The zfALFF pattern included bilateral superior occipital gyrus, right inferior temporal gyrus, left superior frontal gyrus orbital part, right lingual gyrus, left middle occipital gyrus, right precuneus, left cingulate gyrus, left middle temporal gyrus, and left inferior parietal gyrus. The zREHO pattern included bilateral precuneus, bilateral middle temporal gyrus, bilateral caudate, bilateral middle occipital gyrus, bilateral postcentral gyrus, right fusiform gyrus, left inferior temporal gyrus, left inferior frontal gyrus, right middle frontal gyrus, left inferior frontal gyrus orbital part, left middle frontal gyrus orbital part, right superior temporal gyrus, left superior parietal gyrus, left precentral gyrus, right putamen, and right insula. The spatial correlation results across voxel confirmed a high spatial similarity of weights maps between zfALFF and zREHO, but not GMV (Figure S4).

Key features identified for GMV were more prominent within visual, default mode, somatomotor, and dorsal attention networks, while functional discriminative features were primarily located within the default mode and frontoparietal and visual networks (Figure S5).

### 3.4 Multivariate RVR analysis in DS and NDS groups

**FIGURE 4:**
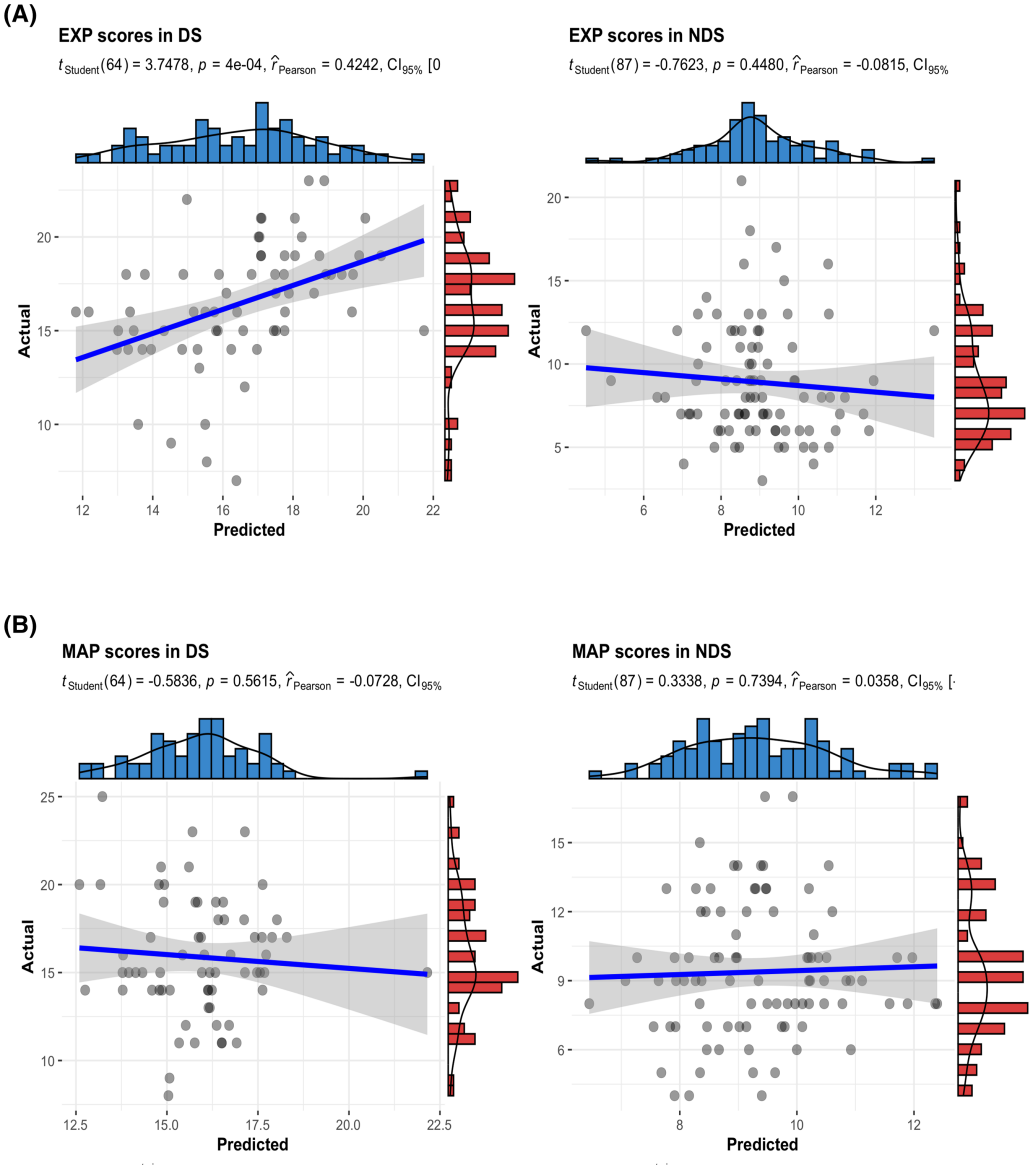
RVR method analysis between the actual scores and predicted scores of negative symptom subdomains in DS and NDS groups, respectively. (A) EXP scores prediction in the DS group; (B) EXP scores prediction in the NDS group; (C) MAP scores prediction in the DS group; (B) MAP scores prediction in the NDS group.

**FIGURE 5:**
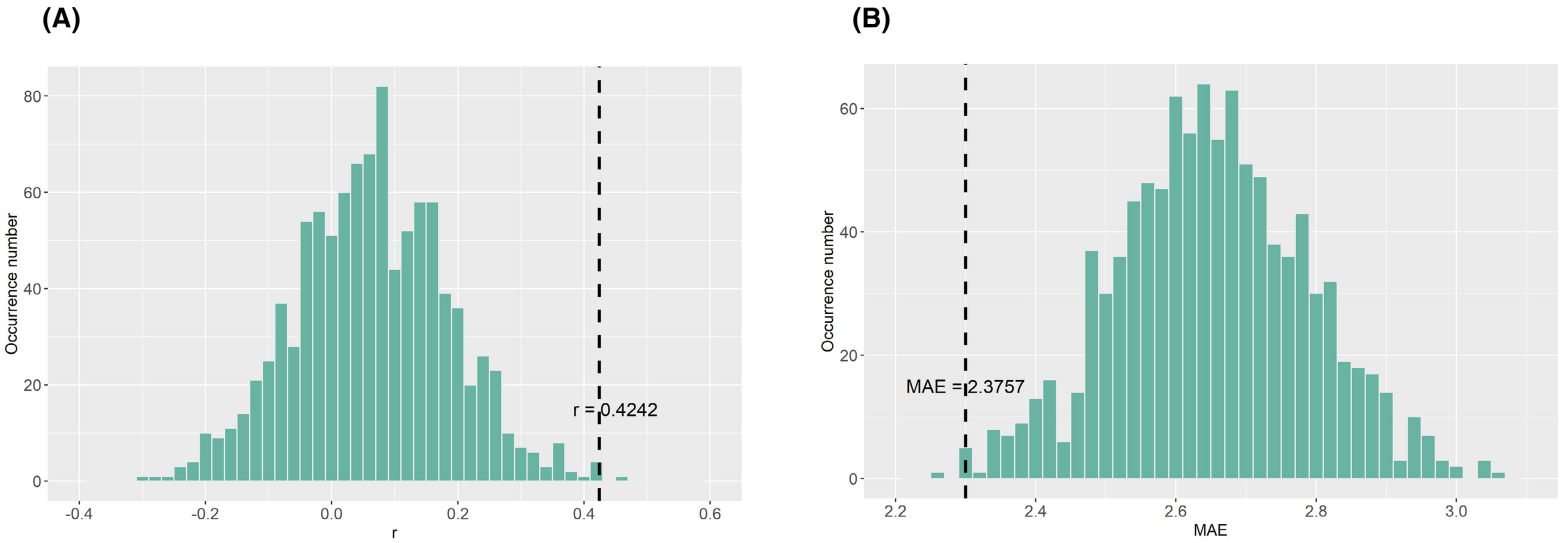
Distribution of permutation test (1000 times) of the prediction *r* (A) and mean absolute error (B), the dashed represented the actual *r*, and MAE observed in RVR performance predicting EXP scores in the DS group.

The results showed a significant positive association (*r* = 0.4242, *p* < 0.001; MAE = 2.3757) between the predicted and actual EXP scores in the DS group, but not in NDS (Figure 4A,B). No significant association was found between predicted and actual MAP scores both in DS and NDS groups (Figure 4C,D). The permutation tests revealed that the *r* value and MAE (*p*_r_ = 0.004, *p*_MAE_ = 0.028) were statistically significant compared to by chance (Figure 5).

### 3.5 Validation analysis

We performed a sensitivity analysis to investigate the influence of disease duration of DS and NDS in our results by adding the variable as a feature in the classification model and found the results remained consistent. The accuracy of subgroups reached 74.84% in the combined classifier (Figure S6).

## 4 DISCUSSION

To the best of our knowledge, the present study is the first, employing multimodal neuroimaging features, alongside machine learning techniques, to investigate the classification of DS and NDS. We demonstrated that the local properties of brain regions extracted from multimodal imaging could distinguish DS from NDS. Our findings provided evidence that the classifier trained by multimodal features achieved a relatively high classification accuracy of 75.48%. Key features identified for GMV were more widespread within visual, default mode, somatomotor, and dorsal attention networks, while both functional features (fALFF and REHO) were identified from the default mode, frontoparietal and visual networks. Notably, based on the top 10% weight, a robust relationship could be predicted by the RVR model in EXP subdomains. These findings indicated that using a combination of multimodal MRI data could improve the neuromarker identification, improving the clinical assessment of deficit syndrome.

In the current study, multimodal classifiers exhibited higher accuracies than unimodal ones in discriminating schizophrenia subtypes or disease conditions, suggesting an advantage of the cross‐information provided by multiple imaging measurements for the distinct characterization of potential biomarkers for the deficit subtype of schizophrenia. Integration of multimodal brain data collected from the same individual could effectively increase the strength of each imaging modality and provide a comprehensive view into the brain for more precise categorical performance or prediction of several important behavioral aspects (e.g., cognition or symptom severity).^32^ Here, the multimodal features included three measures that provide functional information ranging from focal activity (fALFF) to regional coordination (REHO) and structural information (GMV) of the brain, which would be beneficial for a more holistic understanding of heterogeneity in the patients with schizophrenia. The finding that multimodality models outperformed the unimodality models also confirmed our hypothesis that multimodality offers more comprehensive idiosyncrasies in the neural basis of accurate differentiation for schizophrenia subtypes and improves the exploration of potential biomarkers. Moreover, the different contributions for the classification of brain structure and function might reveal a common phenomenon in psychotic disorders that the brain structural and functional abnormalities do not occur simultaneously.

Compared with the previous DS studies, the relatively consistent result is the abnormality in the temporal lobe, which contributed to functional and structural features. Previous researchers have reported a loss of gray matter volume in the right temporal lobe,^33^ reduced cortical thickness in the left temporoparietal junction area,^9^, ^10^, ^34^ and abnormal nodal connectivity in the right inferior temporal gyrus^12^ in the DS. We found that both the functional and structural metrics of the bilateral temporal lobe could provide valuable contribution for distinguish DS patients, suggesting that the temporal lobe is a critical region for deficit syndrome. Interestingly, the temporal poles, including bilateral temporoparietal junction and superior temporal sulcus, are the core components of the functional network mediating the general Theory of Mind (ToM) ability,^35^ which has also been demonstrated to be associated with the severity of negative symptoms in schizophrenia.^36^, ^37^, ^38^, ^39^, ^40^ The structural abnormality in temporal lobes in DS might relate to the early‐onset nonprogressive developmental process, interfering since childhood with the acquisition of basic cognitive and social skills, explaining the poor premorbid adjustment and social cognition observed in this subgroup.^33^ Functional or structural abnormality in the temporal lobe might interfere with the social cognition, with a high impact on the capacity to interact in the social world,^41^ consistent with the core impairment of social functioning in deficit schizophrenia. Additionally, the inferior temporal gyrus region was reported necessary for language formulation,^42^ which might be related to the similar phenomenon for the poverty of speech with negative symptoms. According to our findings, classification results confirmed that the temporal lobe might be impaired in DS both in the brain function and structure, indicating substantial differences between the two subgroups and a potential biomarker for distinguishing DS from NDS using the multimodal features.

We also found the discriminative brain regions not previously reported in the literature, such as the inferior frontal gyrus (IFG), fusiform gyrus, cingulate gyrus, putamen, caudate, and insula. The abnormality of functional and structural metrics in IFG (including the orbital and opercular part) and visual regions contributes to majority of weight for distinguishing the two subtypes. Previous studies have reported the functional disconnections^43^, ^44^, ^45^, ^46^ or volume deficits^47^ of these regions in schizophrenia. Notably, it has been reported that significant deficits of gray matter volume in the anterior cingulated and medial frontal gyrus were found in schizophrenic patients with prominent negative symptoms^48^ or reduced anterior cingulate gray matter volume and thickness in the DS.^49^

The RVR model confirmed discriminative brain features could contribute to the predictions of EXP subdomains in DS. Prior studies have inferred latent structures using exploratory factor analysis, leading to the conclusion that two dimensions reflect MAP and EXP factors.^50^ The EXP subdomain has been investigated less than MAP and has been hypothesized to be related to neurocognitive or social cognition deficits.^51^ Neuroimaging studies investigated the role of clinical measures in evaluating negative symptoms and the relationship between schizophrenia‐related brain abnormalities and clinical measures. Kirschner et al. reported that the MAP factor was negatively correlated with the putamen and nucleus accumbens volumes in patients with schizophrenia.^52^ Amodio et al. found that the MAP was related to the abnormal connection between the left amygdala and the ventral anterior insula, which was known as motivation‐related circuits.^53^ Another research using fMRI to study a model of emotional processing deficits as the neurofunctional basis of blunted affect in schizophrenia showed that patients with flat affect exhibited reduced activation of the parahippocampal gyrus.^54^ Different from previous research, We first used the machine learning method to determine the relationship between brain MRI data and individual negative symptoms, which would provide further insight into the neurobiological mechanisms underlying specific negative symptoms in schizophrenia.^55^ The RVR model successfully predicted EXP factor scores in the DS group, possibly indicating the difference in pathophysiological mechanisms or treatment effects of these two subgroups. Our results are consistent with prior research indicating that the severity of affective flattening showed a significant negative correlation with signal activities in the mirror neuron system, including the premotor cortex, motor cortex, and inferior parietal lobule.^56^ This evidence indicates that the EXP subdomain might be the ‘core symptom’ of primary negative symptoms. Notably, we did not find a significant correlation between symptom scores and the classification model in the NDS group. A potential reason may be that negative symptom subdomain measures show small variations in the non‐deficit schizophrenia individuals compared with DS, which may be too low for the algorithms to pick up on predictive features.

Sample size plays a key role in machine learning. A larger sample would provide greater statistical power and enhance the reliability of findings. However, due to the resource constraints of schizophrenia patients with deficit syndrome and longitudinal assessments for primary negative symptoms,^8^ recruiting a larger sample was challenging. Lee et al.^57^ evaluated the performance of SVMs based on a large single‐site schizophrenia dataset and investigated the effects of training sample size on classification accuracy. They found the classification accuracy of SVMs based on incremental training sample sizes improved consistently from 72.61% to 83.32%, and >81% accuracy was achieved after training sample size >240. Compared with Lee's work, our overall training sample size was 273 (155 schizophrenic patients and 118 healthy controls) and remained relatively moderate when divided into subgroups. From an alternative perspective, the relationship between classification performance and training sample sizes may depend on the features and complexities of algorithms. Prior studies have demonstrated that repeated random splits method should be preferred in a limited sample size.^58^ Accordingly, we performed 10‐fold cross‐validation to estimate the classification generalizability independently and chose the combined structural and functional fMRI features. Additionally, the present study has comparatively the largest sample size in the neuroimaging research of deficit schizophrenia currently, providing empirical insights for future investigations. Moreover, the present study only included male participants, because the existing literature reported a predominance of male patients with deficit schizophrenia.^59^, ^60^ Despite the potential limitations in generalizing to both genders, this deliberate inclusion of exclusively male participants serves to accentuate the unique characteristics of deficit schizophrenia.

### 4.1 Limitations

The present study had several limitations. First, small sample size and single‐center are potential factors that contributed to the inflated accuracies. Therefore, we used 10‐fold cross‐validation to estimate the generalization of the model, which was proved can be applied to small datasets. The current study is preliminary and future studies with large samples and multicenter are warranted. Second, the present findings are based on cross‐sectional data, precluding the extrapolation of longitudinal progression. Therefore, the RVR prediction model between negative symptoms and brain imaging features should be considered correlativity instead of causality. Third, we only used the most common clinical assessments to evaluate symptoms. The two negative symptom domains came from SANS items. A scale that better reflects the latent structure of negative symptoms, such as the Brief Negative Symptoms Scale (BNSS) or Clinical Assessment Interview for Negative Symptoms (CAINS), should be considered in the future. Moreover, we will expand the sample size and use stratified analyses (e.g., age variables) to reduce the effect of nuisance variables in further study.

## 5 CONCLUSIONS

In summary, our findings provided a stable model of combined neuroimaging features that achieved a relatively high classification accuracy for discriminating deficit subgroups. Specifically, machine learning‐based predictive modeling was used to predict subdomains of negative symptom severity from the neuroimaging data. These models can establish the quantitative relationships between symptom scores and brain changes, which can further assist in tracking the progress of neurological diseases and better understanding the pathophysiology of deficit syndrome.

## CONFLICT OF INTEREST STATEMENT

All authors report no biomedical financial interests or potential conflicts of interest.

## Supporting information


Appendix S1

## Data Availability

Research data are not shared.

## References

[1] McGrath J , Saha S , Chant D , Welham J . Schizophrenia: a concise overview of incidence, prevalence, and mortality. Epidemiol Rev. 2008;30:67‐76. 18480098 10.1093/epirev/mxn001

[2] McCutcheon RA , Pillinger T , Mizuno Y , et al. The efficacy and heterogeneity of antipsychotic response in schizophrenia: a meta‐analysis. Mol Psychiatry. 2021;26(4):1310‐1320. 31471576 10.1038/s41380-019-0502-5 PMC7610422

[3] Alnaes D , Kaufmann T , van der Meer D , et al. Brain heterogeneity in schizophrenia and its association with polygenic risk. JAMA Psychiat. 2019;76(7):739‐748. 10.1001/jamapsychiatry.2019.0257 PMC6583664 30969333

[4] Carpenter WT Jr , Heinrichs DW , Wagman AM . Deficit and nondeficit forms of schizophrenia: the concept. Am J Psychiatry. 1988;145(5):578‐583. 3358462 10.1176/ajp.145.5.578

[5] Kirkpatrick B , Fenton WS , Carpenter WT Jr , Marder SR . The NIMH‐MATRICS consensus statement on negative symptoms. Schizophr Bull. 2006;32(2):214‐219. 16481659 10.1093/schbul/sbj053 PMC2632223

[6] Buchanan RW . Persistent negative symptoms in schizophrenia: an overview. Schizophr Bull. 2007;33(4):1013‐1022. 17099070 10.1093/schbul/sbl057 PMC2632326

[7] Kirkpatrick B , Galderisi S . Deficit schizophrenia: an update. World Psychiatry. 2008;7(3):143‐147. 18836581 10.1002/j.2051-5545.2008.tb00181.x PMC2559917

[8] Kirkpatrick B , Buchanan RW , McKenney PD , Alphs LD , Carpenter WT Jr . The schedule for the deficit syndrome: an instrument for research in schizophrenia. Psychiatry Res. 1989;30(2):119‐123. 2616682 10.1016/0165-1781(89)90153-4

[9] Xie T , Zhang X , Tang X , et al. Mapping convergent and divergent cortical thinning patterns in patients with deficit and nondeficit schizophrenia. Schizophr Bull. 2019;45(1):211‐221. 29272543 10.1093/schbul/sbx178 PMC6293229

[10] Fischer BA , Keller WR , Arango C , et al. Cortical structural abnormalities in deficit versus nondeficit schizophrenia. Schizophr Res. 2012;136(1–3):51‐54. 22336954 10.1016/j.schres.2012.01.030 PMC3298625

[11] Takayanagi Y , Sasabayashi D , Takahashi T , et al. Altered brain gyrification in deficit and non‐deficit schizophrenia. Psychol Med. 2019;49(4):573‐580. 29739476 10.1017/S0033291718001228

[12] Yu M , Dai Z , Tang X , et al. Convergence and divergence of brain network dysfunction in deficit and non‐deficit schizophrenia. Schizophr Bull. 2017;43(6):1315‐1328. 29036672 10.1093/schbul/sbx014 PMC5737538

[13] Rozycki M , Satterthwaite TD , Koutsouleris N , et al. Multisite machine learning analysis provides a robust structural imaging signature of schizophrenia detectable across diverse patient populations and within individuals. Schizophr Bull. 2018;44(5):1035‐1044. 29186619 10.1093/schbul/sbx137 PMC6101559

[14] Zeng LL , Wang H , Hu P , et al. Multi‐site diagnostic classification of schizophrenia using discriminant deep learning with functional connectivity MRI. EBioMedicine. 2018;30:74‐85. 29622496 10.1016/j.ebiom.2018.03.017 PMC5952341

[15] Orban P , Dansereau C , Desbois L , et al. Multisite generalizability of schizophrenia diagnosis classification based on functional brain connectivity. Schizophr Res. 2018;192:167‐171. 28601499 10.1016/j.schres.2017.05.027

[16] Viviano JD , Buchanan RW , Calarco N , et al. Resting‐state connectivity biomarkers of cognitive performance and social function in individuals with schizophrenia Spectrum disorder and healthy control subjects. Biol Psychiatry. 2018;84(9):665‐674. 29779671 10.1016/j.biopsych.2018.03.013 PMC6177285

[17] Chen J , Patil KR , Weis S , et al. Neurobiological divergence of the positive and negative schizophrenia subtypes identified on a new factor structure of psychopathology using non‐negative factorization: an International Machine Learning Study. Biol Psychiatry. 2020;87(3):282‐293. 31748126 10.1016/j.biopsych.2019.08.031 PMC6946875

[18] Ma X , Yang WFZ , Zheng W , et al. Neuronal dysfunction in individuals at early stage of schizophrenia, a resting‐state fMRI study. Psychiatry Res. 2023;322:115123. 36827856 10.1016/j.psychres.2023.115123

[19] Gray JP , Muller VI , Eickhoff SB , Fox PT . Multimodal abnormalities of brain structure and function in major depressive disorder: a meta‐analysis of neuroimaging studies. Am J Psychiatry. 2020;177(5):422‐434. 32098488 10.1176/appi.ajp.2019.19050560 PMC7294300

[20] Li Q , Zhao W , Palaniyappan L , Guo S . Atypical hemispheric lateralization of brain function and structure in autism: a comprehensive meta‐analysis study. Psychol Med. 2023;1‐12. doi:10.1017/S0033291723000181 37014101

[21] Harrison TM , Maass A , Adams JN , Du R , Baker SL , Jagust WJ . Tau deposition is associated with functional isolation of the hippocampus in aging. Nat Commun. 2019;10(1):4900. 31653847 10.1038/s41467-019-12921-z PMC6814780

[22] Shang CY , Lin HY , Gau SS . The norepinephrine transporter gene modulates intrinsic brain activity, visual memory, and visual attention in children with attention‐deficit/hyperactivity disorder. Mol Psychiatry. 2021;26(8):4026‐4035. 31595036 10.1038/s41380-019-0545-7

[23] Barendse MEA , Lara GA , Guyer AE , et al. Sex and pubertal influences on the neurodevelopmental underpinnings of schizophrenia: a case for longitudinal research on adolescents. Schizophr Res. 2023;252:231‐241. 36682313 10.1016/j.schres.2022.12.011 PMC10725041

[24] Wang X , Yao S , Kirkpatrick B , Shi C , Yi J . Psychopathology and neuropsychological impairments in deficit and nondeficit schizophrenia of Chinese origin. Psychiatry Res. 2008;158(2):195‐205. 18243336 10.1016/j.psychres.2006.09.007

[25] Cohen AS , Saperstein AM , Gold JM , Kirkpatrick B , Carpenter WT Jr , Buchanan RW . Neuropsychology of the deficit syndrome: new data and meta‐analysis of findings to date. Schizophr Bull. 2007;33(5):1201‐1212. 17159230 10.1093/schbul/sbl066 PMC2632354

[26] Andreasen NC . Negative symptoms in schizophrenia. Definition and reliability. Arch Gen Psychiatry. 1982;39(7):784‐788. 7165477 10.1001/archpsyc.1982.04290070020005

[27] Blanchard JJ , Cohen AS . The structure of negative symptoms within schizophrenia: implications for assessment. Schizophr Bull. 2006;32(2):238‐245. 16254064 10.1093/schbul/sbj013 PMC2632211

[28] Yan CG , Wang XD , Zuo XN , Zang YF . DPABI: Data processing & analysis for (resting‐state) brain imaging. Neuroinformatics. 2016;14(3):339‐351. 27075850 10.1007/s12021-016-9299-4

[29] Zang YF , He Y , Zhu CZ , et al. Altered baseline brain activity in children with ADHD revealed by resting‐state functional MRI. Brain Dev. 2007;29(2):83‐91. 16919409 10.1016/j.braindev.2006.07.002

[30] Zang Y , Jiang T , Lu Y , He Y , Tian L . Regional homogeneity approach to fMRI data analysis. Neuroimage. 2004;22(1):394‐400. 15110032 10.1016/j.neuroimage.2003.12.030

[31] Cui Z , Gong G . The effect of machine learning regression algorithms and sample size on individualized behavioral prediction with functional connectivity features. Neuroimage. 2018;178:622‐637. 29870817 10.1016/j.neuroimage.2018.06.001

[32] Sui J , Jiang R , Bustillo J , Calhoun V . Neuroimaging‐based individualized prediction of cognition and behavior for mental disorders and health: methods and promises. Biol Psychiatry. 2020;88(11):818‐828. 32336400 10.1016/j.biopsych.2020.02.016 PMC7483317

[33] Galderisi S , Quarantelli M , Volpe U , et al. Patterns of structural MRI abnormalities in deficit and nondeficit schizophrenia. Schizophr Bull. 2008;34(2):393‐401. 17728266 10.1093/schbul/sbm097 PMC2632416

[34] Cascella NG , Fieldstone SC , Rao VA , Pearlson GD , Sawa A , Schretlen DJ . Gray‐matter abnormalities in deficit schizophrenia. Schizophr Res. 2010;120(1–3):63‐70. 20452187 10.1016/j.schres.2010.03.039

[35] Bosia M , Riccaboni R , Poletti S . Neurofunctional correlates of theory of mind deficits in schizophrenia. Curr Top Med Chem. 2012;12(21):2284‐2302. 23279170 10.2174/156802612805289917

[36] Pedersen A , Koelkebeck K , Brandt M , et al. Theory of mind in patients with schizophrenia: is mentalizing delayed? Schizophr Res. 2012;137(1–3):224‐229. 22406281 10.1016/j.schres.2012.02.022

[37] Brune M , Lissek S , Fuchs N , et al. An fMRI study of theory of mind in schizophrenic patients with “passivity” symptoms. Neuropsychologia. 2008;46(7):1992‐2001. 18329671 10.1016/j.neuropsychologia.2008.01.023

[38] Benedetti F , Bernasconi A , Bosia M , et al. Functional and structural brain correlates of theory of mind and empathy deficits in schizophrenia. Schizophr Res. 2009;114(1–3):154‐160. 19632816 10.1016/j.schres.2009.06.021

[39] Lee J , Quintana J , Nori P , Green MF . Theory of mind in schizophrenia: exploring neural mechanisms of belief attribution. Soc Neurosci. 2011;6(5–6):569‐581. 22050432 10.1080/17470919.2011.620774 PMC3928144

[40] Brune M , Ozgurdal S , Ansorge N , et al. An fMRI study of “theory of mind” in at‐risk states of psychosis: comparison with manifest schizophrenia and healthy controls. Neuroimage. 2011;55(1):329‐337. 21147235 10.1016/j.neuroimage.2010.12.018

[41] Brune M , Brune‐Cohrs U . Theory of mind – evolution, ontogeny, brain mechanisms and psychopathology. Neurosci Biobehav Rev. 2006;30(4):437‐455. 16239031 10.1016/j.neubiorev.2005.08.001

[42] Dien J , Brian ES , Molfese DL , Gold BT . Combined ERP/fMRI evidence for early word recognition effects in the posterior inferior temporal gyrus. Cortex. 2013;49(9):2307‐2321. 23701693 10.1016/j.cortex.2013.03.008 PMC3758432

[43] Lynall ME , Bassett DS , Kerwin R , et al. Functional connectivity and brain networks in schizophrenia. J Neurosci. 2010;30(28):9477‐9487. 20631176 10.1523/JNEUROSCI.0333-10.2010 PMC2914251

[44] Fornito A , Yoon J , Zalesky A , Bullmore ET , Carter CS . General and specific functional connectivity disturbances in first‐episode schizophrenia during cognitive control performance. Biol Psychiatry. 2011;70(1):64‐72. 21514570 10.1016/j.biopsych.2011.02.019 PMC4015465

[45] Backasch B , Sommer J , Klohn‐Saghatolislam F , Muller MJ , Kircher TT , Leube DT . Dysconnectivity of the inferior frontal gyrus: implications for an impaired self‐other distinction in patients with schizophrenia. Psychiatry Res. 2014;223(3):202‐209. 24976632 10.1016/j.pscychresns.2014.05.007

[46] Reckless GE , Andreassen OA , Server A , Ostefjells T , Jensen J . Negative symptoms in schizophrenia are associated with aberrant striato‐cortical connectivity in a rewarded perceptual decision‐making task. Neuroimage Clin. 2015;8:290‐297. 26106553 10.1016/j.nicl.2015.04.025 PMC4474284

[47] Ohtani T , Levitt JJ , Nestor PG , et al. Prefrontal cortex volume deficit in schizophrenia: a new look using 3T MRI with manual parcellation. Schizophr Res. 2014;152(1):184‐190. 24280350 10.1016/j.schres.2013.10.026

[48] Sigmundsson T , Suckling J , Maier M , et al. Structural abnormalities in frontal, temporal, and limbic regions and interconnecting white matter tracts in schizophrenic patients with prominent negative symptoms. Am J Psychiatry. 2001;158(2):234‐243. 11156806 10.1176/appi.ajp.158.2.234

[49] Takayanagi M , Wentz J , Takayanagi Y , et al. Reduced anterior cingulate gray matter volume and thickness in subjects with deficit schizophrenia. Schizophr Res. 2013;150(2–3):484‐490. 24035178 10.1016/j.schres.2013.07.036 PMC4076020

[50] Strauss GP , Nunez A , Ahmed AO , et al. The latent structure of negative symptoms in schizophrenia. JAMA Psychiat. 2018;75(12):1271‐1279. 10.1001/jamapsychiatry.2018.2475 PMC6583036 30208377

[51] Marder SR , Galderisi S . The current conceptualization of negative symptoms in schizophrenia. World Psychiatry. 2017;16(1):14‐24. 28127915 10.1002/wps.20385 PMC5269507

[52] Kirschner M , Schmidt A , Hodzic‐Santor B , et al. Orbitofrontal‐striatal structural alterations linked to negative symptoms at different stages of the schizophrenia spectrum. Schizophr Bull. 2021;47(3):849‐863. 33257954 10.1093/schbul/sbaa169 PMC8084448

[53] Amodio A , Quarantelli M , Mucci A , et al. Avolition‐apathy and white matter connectivity in schizophrenia: reduced fractional anisotropy between amygdala and insular cortex. Clin EEG Neurosci. 2018;49(1):55‐65. 29243529 10.1177/1550059417745934

[54] Lepage M , Sergerie K , Benoit A , Czechowska Y , Dickie E , Armony JL . Emotional face processing and flat affect in schizophrenia: functional and structural neural correlates. Psychol Med. 2011;41(9):1833‐1844. 21284912 10.1017/S0033291711000031

[55] Morch‐Johnsen L , Agartz I , Jensen J . The neural correlates of negative symptoms in schizophrenia: examples from MRI literature. Clin EEG Neurosci. 2018;49(1):12‐17. 29243527 10.1177/1550059417746214

[56] Lee JS , Chun JW , Yoon SY , Park HJ , Kim JJ . Involvement of the mirror neuron system in blunted affect in schizophrenia. Schizophr Res. 2014;152(1):268‐274. 24268934 10.1016/j.schres.2013.10.043

[57] Lee LH , Chen CH , Chang WC , et al. Evaluating the performance of machine learning models for automatic diagnosis of patients with schizophrenia based on a single site dataset of 440 participants. Eur Psychiatry. 2021;65(1):e1. 34937587 10.1192/j.eurpsy.2021.2248 PMC8792868

[58] Varoquaux G , Raamana PR , Engemann DA , Hoyos‐Idrobo A , Schwartz Y , Thirion B . Assessing and tuning brain decoders: cross‐validation, caveats, and guidelines. Neuroimage. 2017;145(Pt B):166‐179. 27989847 10.1016/j.neuroimage.2016.10.038

[59] Kirkpatrick B , Ross DE , Walsh D , Karkowski L , Kendler KS . Family characteristics of deficit and nondeficit schizophrenia in the Roscommon Family Study. Schizophr Res. 2000;45(1–2):57‐64. 10978873 10.1016/s0920-9964(99)00164-4

[60] Arango C , Bobes J , Kirkpatrick B , Garcia‐Garcia M , Rejas J . Psychopathology, coronary heart disease and metabolic syndrome in schizophrenia spectrum patients with deficit versus non‐deficit schizophrenia: findings from the CLAMORS study. Eur Neuropsychopharmacol. 2011;21(12):867‐875. 21477998 10.1016/j.euroneuro.2011.03.005

