# Supplementary material for: A neuromarker for deficit syndrome in schizophrenia from a combination of structural and functional magnetic resonance imaging
Source: CNS Neurosci Ther. 2023 Jun 8;29(12):3774–85. doi: 10.1111/cns.14297 (PMC10651988; doi:10.1111/cns.14297)
Supplement: Supplementary file 1 — Appendix S1 [file CNS-29-3774-s001.docx]

Supplementary Information

**1 Methods**

**1.1 Samples**

The subjects of this study were all male patients with schizophrenia who were clinically stable in the inpatient department of Wutaishan Hospital in Yangzhou, Jiangsu Province from 2016 to 2021, and age-sex-matched normal control subjects were recruited during the same period. The researcher explained the purpose and significance of this study to all subjects and their legal guardians, and after obtaining consent, the subjects themselves or their legal guardians signed an informed consent form. This study was approved by the Ethics Committee of Nanjing Brain Hospital and the Ethics Committee of Yangzhou Wutaishan Hospital.

Patient group inclusion criteria: (1) Meet the diagnostic criteria for schizophrenia in the fourth edition of the Diagnostic and Statistical Manual of Mental Disorders (DSM-IV-TR), verified by the Structured Clinical Interview for DSM-IV (SCID-I); (2) Male, right-handed, Chinese Han population; (3) Aged between 20-65 years old; (4) Long-term stable mental symptoms, taking fixed antipsychotic drugs for at least 12 months (Checked the information via hospital records).

Exclusion criteria for patient group: (1) Brain organic diseases, such as: brain tumor, brain trauma, mental retardation, epilepsy; (2) History of heart, liver, kidney, endocrine and hematopoietic system diseases that seriously affect cognitive function; (3) History of psychoactive substance abuse, history of dependence on alcohol, drugs, etc; (4) History of electroconvulsive therapy within the past 6 months; (5) Those who are contraindicated in magnetic resonance scanning.

Patients with schizophrenia who met the above-mentioned inclusion and exclusion criteria were further screened by two attending physicians for subtypes of deficient schizophrenia, using the Schedule for the Deficit Syndrome (SDS) Chinese scale tool. Edition [^1^](#_ENREF_1).

Inclusion criteria for normal control group: (1) Age and education level should be matched with the schizophrenia patient group as much as possible; (2) Male, right-handed, Chinese Han population; (3) Aged between 20-65 years old; (4) There was no obvious abnormality in the scores of neurocognitive scales.

Exclusion criteria for normal control group: (1) Positive family history of mental illness; (2) History of organic diseases such as neurodegenerative diseases, brain trauma, brain tumors, and cerebrovascular diseases; (3) History of major physical diseases such as severe heart, liver, and renal insufficiency; (4) Have a history of substance dependence or abuse in the past; (5) Those who are contraindicated in magnetic resonance scanning.

**1.2 Clinical Assessments**

The sociodemographic data of the research subjects, including name, gender, age, education level, marital status, occupation, smoking and other information, were collected using a general situation questionnaire.

The enrolled patients were clinically assessed by two senior attending physicians who were trained in consistent assessment. (1) Schedule for the Deficit Syndrome (SDS) The subtypes of the subjects who meet the diagnostic criteria of DSM-IV-TR for schizophrenia are classified into subtypes, and they are divided into the deficient schizophrenia (DS) group and the non-deficient schizophrenia (NDS) group according to the Chinese version of SDS [^1^](#_ENREF_1). The scale has good reliability and validity in the Chinese schizophrenia population.

**SDS**

The specific diagnostic criteria of the SDS scale are as follows:

1. Symptomatic criteria: including 6 negative symptom clusters (0-4 points, 5-point scale): (1) limited affect; (2) reduced affective range; (3) poor speech; (4) inhibited interest; (5) Poor sense of purpose; (6) Decreased social motivation. The above symptom groups are further classified into two factors: emotion expression factor (1-3) and lack of motivation factor (4-6);

2. Severity and disease course criteria: the above two or more negative symptoms have reached clinical significance (score ≥ 2) and have persisted for 12 months, and persisted in the clinical stable period; 3. The above symptoms are primary or idiopathic, not secondary to depression, anxiety, drug side effects, hallucinations, delusions or mental retardation;

**BPRS**

Brief Psychiatric Rating Scale (BPRS) BPRS was used to evaluate the overall mental symptoms, with a total of 18 items, which were subdivided into negative symptoms, positive symptoms, dissociative symptoms and affective symptoms according to the previous comprehensive factor analysis model [^2^](#_ENREF_2)^,^[^3^](#_ENREF_3).

**SANS**

Because the BPRS scale cannot fully reflect the negative symptom factors of all dimensions, SANS is further used to evaluate the negative symptoms of patients [^4^](#_ENREF_4)^,^[^5^](#_ENREF_5). The scale has a total of 19 items and 5 subscales. As shown in Table S1, all SANS-19 items were significantly higher in DS expect inattentiveness items. The evaluation method of negative symptom structural factors, SANS negative symptoms are divided into two sub-domains: motivation and pleasure (MAP) and diminished expressivity (EXP). Inattentiveness items (Work inattentiveness and Inattentiveness during mental status testing) were the least correlated with other items in SANS-19 (Figure S1), so in the data analysis of the scale, we mainly studied the two subdomains of decreased expression and lack of social motivation.

According to Galderisi’s review [^6^](#_ENREF_6), the MAP and EXP score were calculated as follow:

EXP = sum SANS item 1, 2, 3, 4, 5, 6, 7;

MAP = sum SANS item 11, 12, 13, 14, 15, 16, 17.

**SAPS**

The positive symptoms of patients were evaluated by SAPS, with a total of 34 items and a 6-level score.

**1.3 Imaging measures**

**scanner parameters**

The resting-state fMRI images were acquired using a gradient-recalled echo echo-planar imaging sequence under the following parameters: repetition time = 2000 ms; echo time = 25 ms; flip angle = 90°; field of view = 240×240 mm^2^; matrix size = 64×64; thickness = 4 mm without gap; voxel size = 4.0×4.0×4.0 mm^3^; slice number = 35; and number of time points = 240. The T1 weighted structural images were acquired using three-dimensional brain volume imaging sequence with high resolution under the following parameters: TR = 11.94 ms; TE = 5.044 ms; flip angle = 15°; FOV = 240 × 240 mm; matrix size = 256×256; slice thickness = 1 mm without gap; voxel size = 1×1×1 mm^3^; slice number = 172.

**Gray matter volume (GMV) calculation**

Voxel-based morphometry (VBM) of computational anatomy analysis was employed using the CAT12 toolbox (<http://dbm.neuro.uni-jena.de/cat12>) running within SPM12 (<http://www.fil.ion.ucl.ac.uk/spm>). All acquired scans were underwent an internal quality protocol in accordance with the recommended parameters of CAT12. In contrast with the original segmentation approach of SPM12, CAT12 is based on an Adaptive Maximum A Posterior (AMAP) technique without the need for a priori information about the tissue probabilities [^7^](#_ENREF_7). The segmentation approach uses a Partial Volume Estimation (PVE) with a simplified mixed model of at most two tissue types [^8^](#_ENREF_8). Each participant’s original T1 image was segmented into three pure classes: gray matter (GM), white matter (WM), and cerebrospinal fluid (CSF) based on the above described AMAP estimation. This results in an estimation of the amount of each pure tissue type presented in every voxel and provided a more accurate segmentation. The segmented images above were used to create a customized template based on all participants by the Diffeomorphic Anatomical Registration Through Exponentiated Lie algebra (DARTEL) technique [^9^](#_ENREF_9). According to this customized template, each subject’s gray matter density (GMD) map was warped, and the resultant image was affine-registered in the Montreal Neurological Institute (MNI) space and resampled to a voxel size of 2×2×2-mm cubic voxel. The gray matter volume (GMV) maps were generated by multiplying the GMD maps with the nonlinear components of the Jacobian determinant and smoothed using an FWHM Gaussian kernel of 8 mm. To create a GM mask, we smoothed GMD maps by averaging all the subjects’ resultant GMD maps and applied a threshold of 0.2 to this average map [^10-12^](#_ENREF_10). All the GMV features were restricted to this GM mask.

**Fractional amplitude of** **low-frequency fluctuations (fALFF) calculation**

Since the low-frequency fluctuations between 0.01 Hz and 0.08 Hz are of particular relevance to the resting-state fMRI [^13^](#_ENREF_13), the fractional amplitude of low-frequency fluctuations (fALFF) was calculated to examine regional spontaneous brain activity [^14^](#_ENREF_14). After preprocessing, the time series for each voxel was filtered (bandpass, 0.01–0.08 Hz) to remove the effects of low-frequency drift and high-frequency noises. The filtered time series were transformed to a frequency domain with a fast Fourier transform (FFT), and the power spectrum was then obtained. ALFF was obtained by summing the amplitudes in the low-frequency band; afterward, fALFF was calculated as ALFF/amplitude of the total frequency. For standardization purposes, the fALFF value of each voxel was z-normalized across the whole brain for each subject.

**Regional homogeneity (REHO) calculation**

We calculated individual REHO maps by computing Kendall’s concordance coefficient (KCC), which measures the BOLD time series for each voxel and the nearest 26 contiguous voxels [^15^](#_ENREF_15) and is calculated as follows:

*W* = $\frac{\sum\left( R_{i} \right)^{2}-n\left( \bar{R} \right)^{2}}{\frac{1}{12}K^{2}\left( n^{3}-n \right)}$

where *W* is the KCC among given voxels, ranging from 0 to 1, *Ri* is the sum rank of the *i*th volumes, $\overline{R}$= ((*n* + 1) *K*)/2 is the mean of the *Ri*’s; *K* is the number of time series within a measured cluster (one given voxel plus the number of its neighbors. Here, we defined K = 27, and *n* is the number of ranks.” [^15^](#_ENREF_15) After calculation, the KCC map of each individual was then z-normalized by dividing KCC in each voxel by the mean KCC of the total gray matter. A 4-mm full-width at half-maximum (FWHM) Gaussian kernel was used for smoothing the zREHO map.

**SVM classification model**

The classifier was designed using a linear support vector machine (SVM, <http://www.csie.ntu.edu.tw/cjlin/libsvm/>) classification model and employed multimodal voxel-based imaging features (GMV, zfALFF, and zREHO). This algorithm minimizes empirical classification errors by taking into account the complexity of the model, which identifies the hyperplane with the maximum margin.[^11^](#_ENREF_11) For controlling the trade-off between empirical classification errors and the complexity, the parameter C was set at the default value (C = 1). Single metric and combined metrics were trained respectively to obtain the decision value of each model. Each voxel in the GMV (*N*_voxel_ = 186589), zfALFF (*N*_voxel_ = 54109), and zREHO (*N*_voxel_ = 54109) maps was extracted to generate a feature vector for classification. Hence, for each participant, there were 186589 GMV features, 54109 zfALFF features, and 54109 zREHO features.

**Networks distribution for discriminative feature**

The GMV, zfALFF, and zREHO top features were extracted and mapped onto a parcellation map of the cerebral cortex that was generated for 7 networks and the subcortical network based on Thomas Yeo’s study [^16^](#_ENREF_16). The values of the discriminative feature weight were calculated in corresponding networks.

**RVR model**

Set in a fully probabilistic Bayesian framework, RVR is a sparse kernel machine-learning algorithm used to predict behavioral measures with resting-state brain data. The Pearson’s correlation coefficient (*r*) and mean absolute error (MAE, formula: $\frac{1}{n}\sum_{i=1}^{n} \left| y_{i}-\hat{y_{i}} \right|,$ *n* indicates sample size, *y_i_* indicates the actual score of the *i^th^* subject, and $\hat{y_{i}}$ indicates the predicted score of the *i^th^* subject) between the observed and predicted scores from DS and NDS groups, respectively.

1. **Results**

2.1 There were significant differences in each items except work inattentiveness and inattentiveness between DS and NDS patients (Table S1). Correlation heatmap showed relatively lower correlation coefficient between inattentiveness items and other subdomains of negative symptoms.

**Table S1**. Comparison of SANS-19 items between DS and NDS

|  | SANS-19 | DS | NDS |
| --- | --- | --- | --- |
| 1 | Unchanging Facial Expression | 2.67 ± 0.79^*^ | 1.47 ± 0.74 |
| 2 | Decreased Spontaneous Movements | 2.14 ± 0.76^*^ | 1.08 ± 0.80 |
| 3 | Paucity of Expressive Gestures | 2.11 ± 0.68^*^ | 1.40 ± 0.84 |
| 4 | Poor Eye Contact | 2.06 ± 0.78^*^ | 0.97 ± 0.85 |
| 5 | Affective Nonresponsivity | 2.52 ± 0.79^*^ | 1.34 ± 0.77 |
| 6 | Lack of Vocal Inflections | 2.35 ± 0.90^*^ | 1.19 ± 0.72 |
| 7 | Poverty of Speech | 2.62 ± 0.76^*^ | 1.46 ± 0.59 |
| 8 | Poverty of content of speech | 2.14 ± 0.80^*^ | 1.38 ± 0.70 |
| 9 | Blocking | 1.74 ± 1.17^*^ | 1.01 ± 0.85 |
| 10 | Increased Latency of Response | 1.82 ± 0.96^*^ | 0.96 ± 0.80 |
| 11 | Grooming and Hygiene | 2.14 ± 0.88^*^ | 1.18 ± 0.67 |
| 12 | Imperesistence at work or school | 2.36 ± 0.97^*^ | 1.65 ± 0.76 |
| 13 | Physical anergia | 2.29 ± 0.86^*^ | 1.37 ± 0.70 |
| 14 | Recreational interests and activities | 2.71 ± 0.70^*^ | 1.42 ± 0.74 |
| 15 | Sexual interest and activity | 1.97 ± 0.98^*^ | 1.58 ± 0.77 |
| 16 | Ability to fell intimacy and closeness | 2.27 ± 0.80^*^ | 1.04 ± 0.78 |
| 17 | Relationships with friends and peers | 2.14 ± 0.63^*^ | 1.13 ± 0.74 |
| 18 | Work inattentiveness | 1.64 ± 1.4 | 1.53 ± 0.88 |
| 19 | Inattentiveness during mental status testing | 1.58 ± 1.55 | 1.30 ± 1.01 |

^*^*p* < 0.001


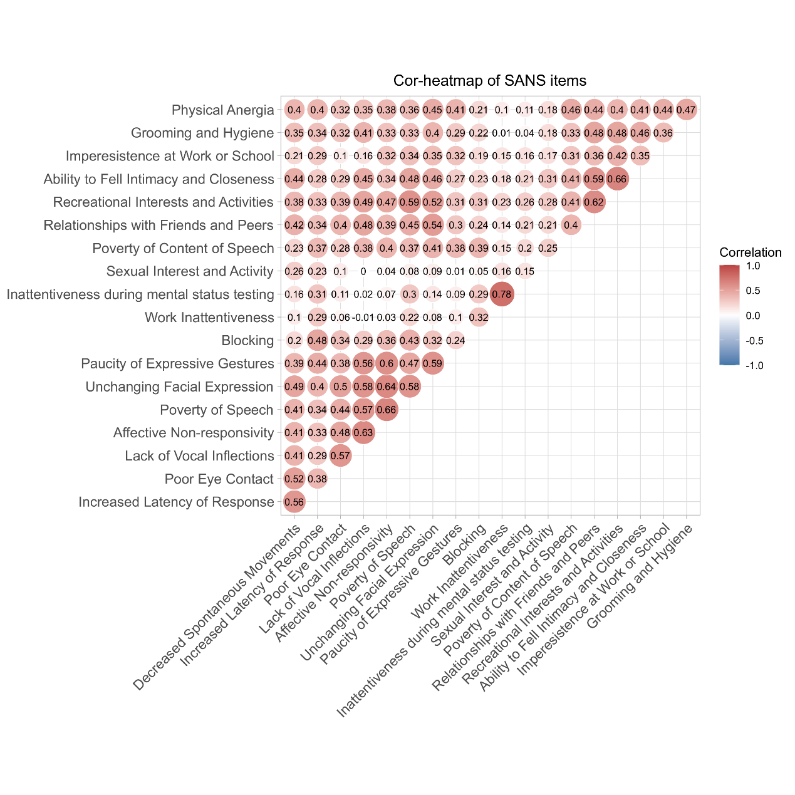


Figure S1. Correlation heatmap of SANS-19 item in all schizophrenia subjects.

2.2 The brain regions that contributed to most classifications between DS and NDS were identified by setting the threshold to > 10% of the maximum weight vector value in combined features, with cluster setting of size of 2,400 mm^3^ of each metric (Table S2).

**Table S2**. Discrimination weights regions (Top 10%) between DS and NDS group of combined-classifier (GMV, zfALFF and zREHO)

| Region | Hemisphere | Cluster size (voxels) | Peak MNI coordinate | | | Weights |
| --- | --- | --- | --- | --- | --- | --- |
|  |  |  | x | y | z |  |
| GMV metric (2 mm cubic voxel) | |  |  |  |  |  |
| Inferior frontal gyrus, orbital part (ORBinf) | L | 564 | -16 | 14 | -28 | 0.00626 |
| Temporal pole, superior temporal gyrus (TPOsup) | L |  |  |  |  |  |
| Inferior frontal gyrus, orbital part (ORBinf) | R | 537 | 18 | 20 | -28 | 0.00577 |
| Temporal pole, superior temporal gyrus (TPOsup) | R |  |  |  |  |  |
| Fusiform gyrus (FFG) | L | 302 | -36 | -78 | -14 | -0.00792 |
| Parahippocampal gyrus (PHG) | R | 612 | 14 | -34 | -12 | -0.00799 |
| Cerebellum lobule IV/V | R |  |  |  |  |  |
| Inferior temporal gyrus (ITG) | L | 331 | -50 | -44 | -8 | -0.00675 |
| Superior frontal gyrus, medial orbital (ORBsupmed) | L | 300 | -8 | 64 | -10 | -0.00522 |
| Superior frontal gyrus, orbital part (ORBsup) | L |  |  |  |  |  |
| Middle occipital gyrus (MOG) | L | 810 | -46 | -76 | 16 | 0.00626 |
| Middle temporal gyrus (MTG) | L |  |  |  |  |  |
| Inferior frontal gyrus, opercular part (IFGoper) | R | 396 | 46 | 4 | 2 | 0.00533 |
| Rolandic operculum (ROL) | R |  |  |  |  |  |
| Postcentral gyrus (PoCG) | L | 414 | -34 | -36 | 64 | -0.00641 |
| Precentral gyrus (PreCG) | R | 333 | 24 | -14 | 76 | 0.00538 |
| Precentral gyrus (PreCG) | L | 325 | -18 | -8 | 62 | -0.00720 |
| Supplementary motor area | L |  |  |  |  |  |
| zfALFF metric (3 mm cubic voxel) | |  |  |  |  |  |
| Inferior temporal gyrus (ITG) | R | 111 | 42 | -3 | -48 | -0.00740 |
| Superior frontal gyrus, orbital part (ORBsup) | L | 192 | -9 | 66 | -3 | -0.00785 |
| Middle frontal gyrus, orbital part (ORBmid) | L |  |  |  |  |  |
| Superior frontal gyrus, medial (SFGmed) | L |  |  |  |  |  |
| Lingual gyrus (LING) | R | 134 | 24 | -96 | -15 | -0.00639 |
| Superior occipital gyrus (SOG) | R |  |  |  |  |  |
| Middle occipital gyrus (MOG) | L | 102 | -18 | -81 | 18 | 0.00691 |
| Superior occipital gyrus (SOG) | L |  |  |  |  |  |
| Precuneus (PCUN) | R | 155 | 9 | -42 | 36 | 0.00639 |
| Median cingulate gyrus (DCG) | R |  |  |  |  |  |
| Middle cingulate gyrus (MCG) | L | 146 | 0 | -6 | 33 | 0.00759 |
| Anterior cingulate gyrus (ACG) | L |  |  |  |  |  |
| Posterior cingulate gyrus (PCG) | L |  |  |  |  |  |
| Middle temporal gyrus (MTG) | L | 89 | -57 | -60 | 30 | 0.00759 |
| Inferior parietal gyrus (IPG) | L | 112 | -54 | -45 | 36 | 0.00810 |
| zREHO metric (3 mm cubic voxel) | |  |  |  |  |  |
| Fusiform (FFG) | R | 586 | 30 | -51 | -15 | -0.00981 |
| Inferior temporal gyrus (ITG) | L | 167 | -54 | -66 | -18 | -0.00895 |
| Inferior frontal gyrus, orbital part (ORBinf) | L | 216 | -39 | 36 | 6 | 0.00718 |
| Middle frontal gyrus, orbital part (ORBmid) | L | 128 | -9 | 54 | -9 | 0.00657 |
| Middle occipital gyrus (MOG) | L | 252 | -18 | -81 | -6 | -0.00737 |
| Inferior frontal gyrus, triangular part (IFGtri) | L | 411 | -51 | 33 | 24 | 0.00839 |
| Inferior frontal gyrus, opercular part (IFGoper) | L |  |  |  |  |  |
| Middle frontal gyrus (MFG) | R | 660 | 39 | 39 | 21 | 0.00988 |
| Inferior frontal gyrus, triangular part (IFGtri) | L |  |  |  |  |  |
| Middle occipital gyrus (MOG) | L | 304 | -36 | -78 | 3 | 0.00803 |
| Middle temporal gyrus (MTG) | L |  |  |  |  |  |
| Precuneus (PCUN) | L | 594 | 3 | -78 | 42 | 0.00840 |
| Precuneus (PCUN) | R |  |  |  |  |  |
| Middle temporal gyrus (MTG) | R | 169 | 51 | -24 | -9 | -0.00778 |
| Superior temporal gyrus (STG) | R |  |  |  |  |  |
| Putamen (PUT) | R | 370 | 45 | -15 | 21 | -0.00797 |
| Insula (INS) | R |  |  |  |  |  |
| Caudate (CAU) | R |  |  |  |  |  |
| Caudate (CAU) | L | 103 | -15 | 15 | 12 | -0.00659 |
| Postcentral gyrus (PoCG) | R | 481 | 27 | -39 | 60 | -0.00979 |
| Middle occipital gyrus (MOG) | R |  |  |  |  |  |
| Superior parietal gyrus (SPG) | L | 138 | -27 | -75 | 33 | -0.00635 |
| Supramarginal gyrus (SMG) | R | 224 | 51 | -45 | 27 | 0.00685 |
| Precentral gyrus (PreCG) | L | 155 | -45 | -15 | 54 | 0.00616 |
| Postcentral gyrus (PoCG) | L |  |  |  |  |  |
| Precuneus (PCUN) | R | 158 | 9 | -27 | 51 | -0.00756 |

2.3 The performance of comparison for 101 times 10-fold cross-validation accuracy results between DS/NDS and HC (Figure S2 & S3), which showed that all the models provided an excellent discriminative pattern (ACC = 84.0–93.48%, AUC = 0.90–0.99), and the combined models generally outperformed the single-modality models


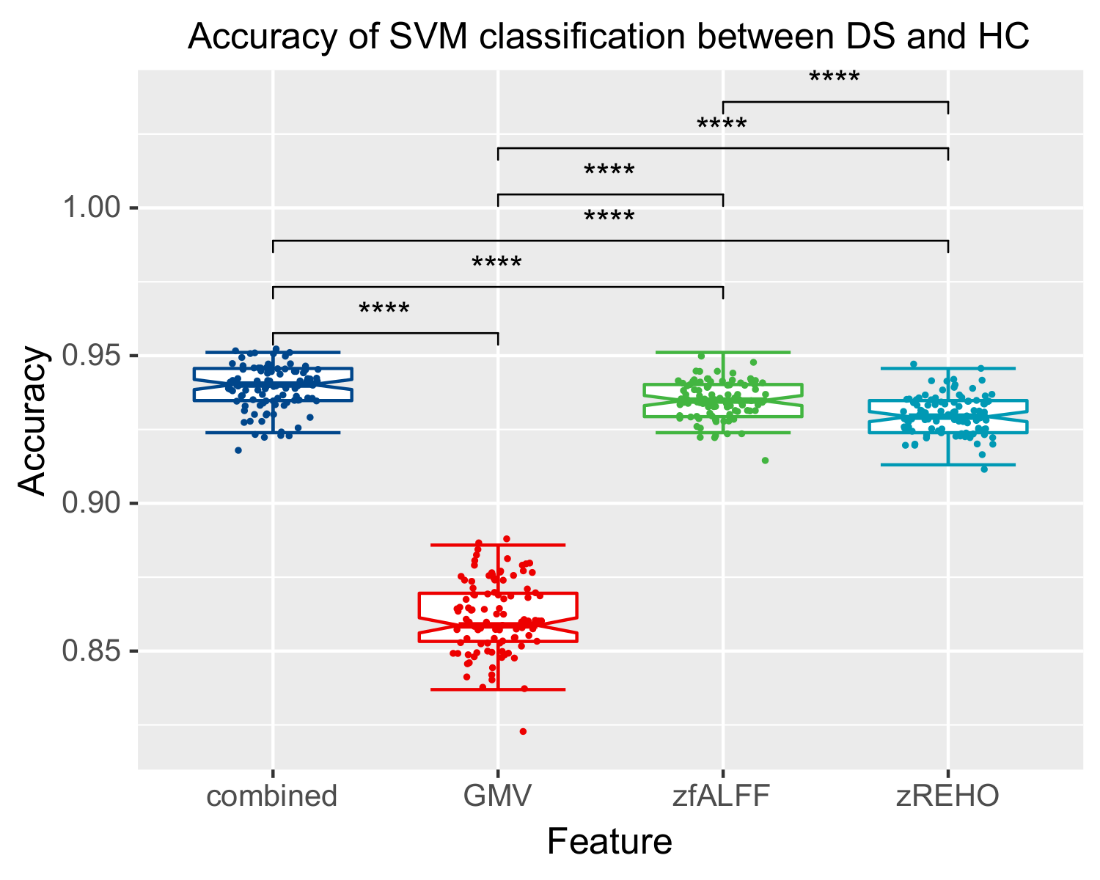


Figure S2. Comparison of accuracy of SVM classification between DS and HC groups (101 times 10-fold cross-validation results).


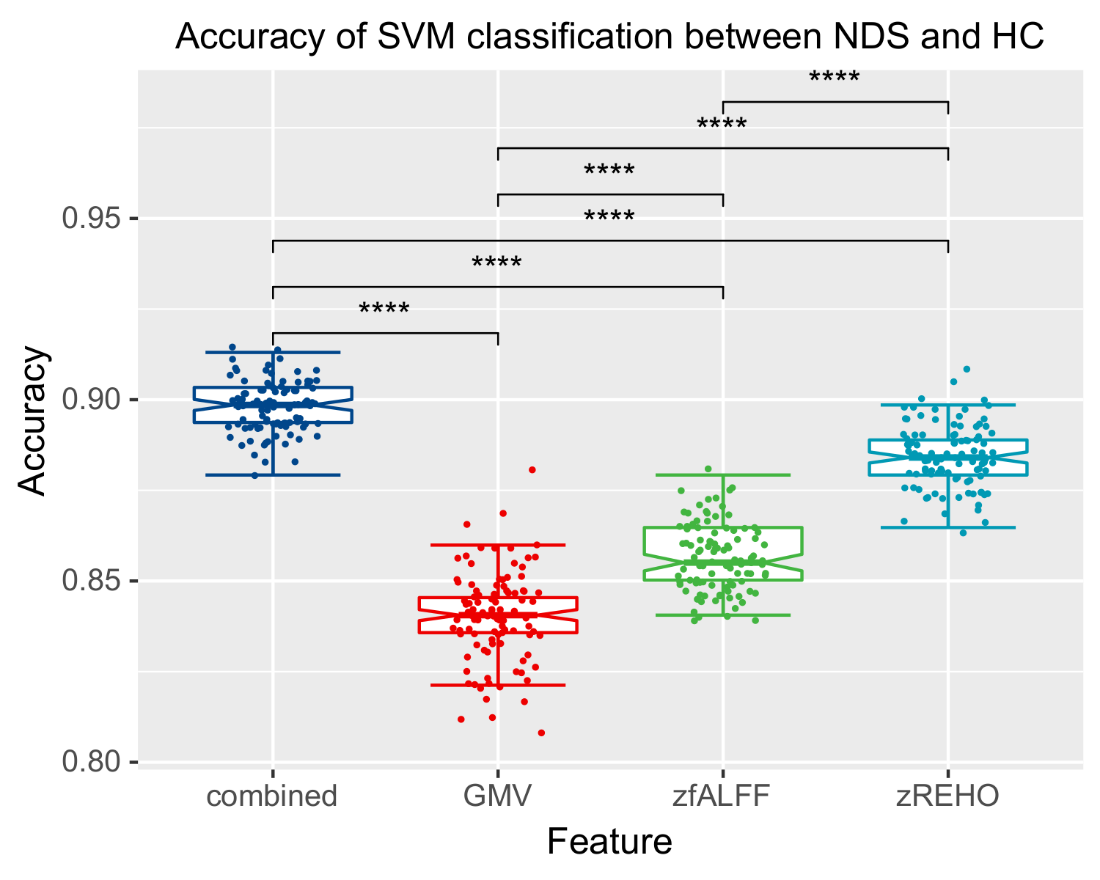


Figure S3. Comparison of accuracy of SVM classification between NDS and HC groups (101 times 10-fold cross-validation results).

2.4 We calculated the spatial correlation between three modality features’ weight. In order to match the voxel number, the GMV weight map was resampled to a voxel size of 3×3×3 mm cubic. The correlation results across voxel confirmed the significant spatial similarity of weights maps between zfALFF and zREHO modality (*r* = 0.3645, *p* < 0.001). However, the spatial similarities between GMV and zfALFF or zREHO weights were low (Figure S4). The top 10% features of the classification model between DS and NDS were mapped into the network module according to the corresponding atlas[^16^](#_ENREF_16) in each modality for better visualization and explanation. Here we used radar chart to show the percentage of each network distribution (Figure S5). Concerning GMV, the most contributive regions were distributed within visual, default mode, somatomotor, and dorsal attention networks (total = 61.58%). Concerning zfALFF and zREHO, the most contributive regions were in default mode, frontoparietal and visual networks (zfALFF total = 52.61%, zREHO total = 52.85%).

1. **Validation analysis**

The accuracy of subgroups reached 74.84% in combined classifier, 69.03% in GMV classifier, 66.45% in zfALFF classifier and 63.23% in zREHO classifier (Figure S6).


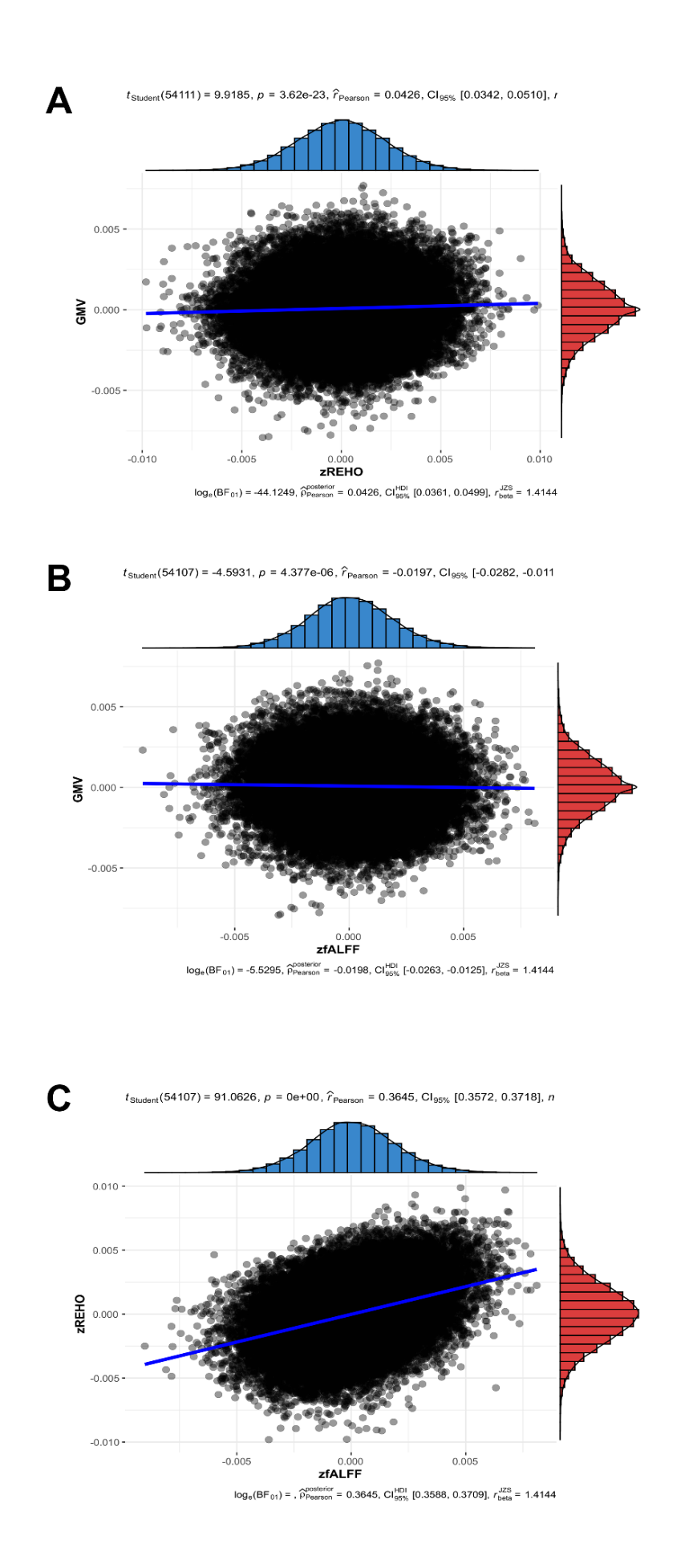


Figure S4. Spatial correlation analysis among GMV, zfALFF and zREHO modality weights according to combined-classifier between DS and NDS


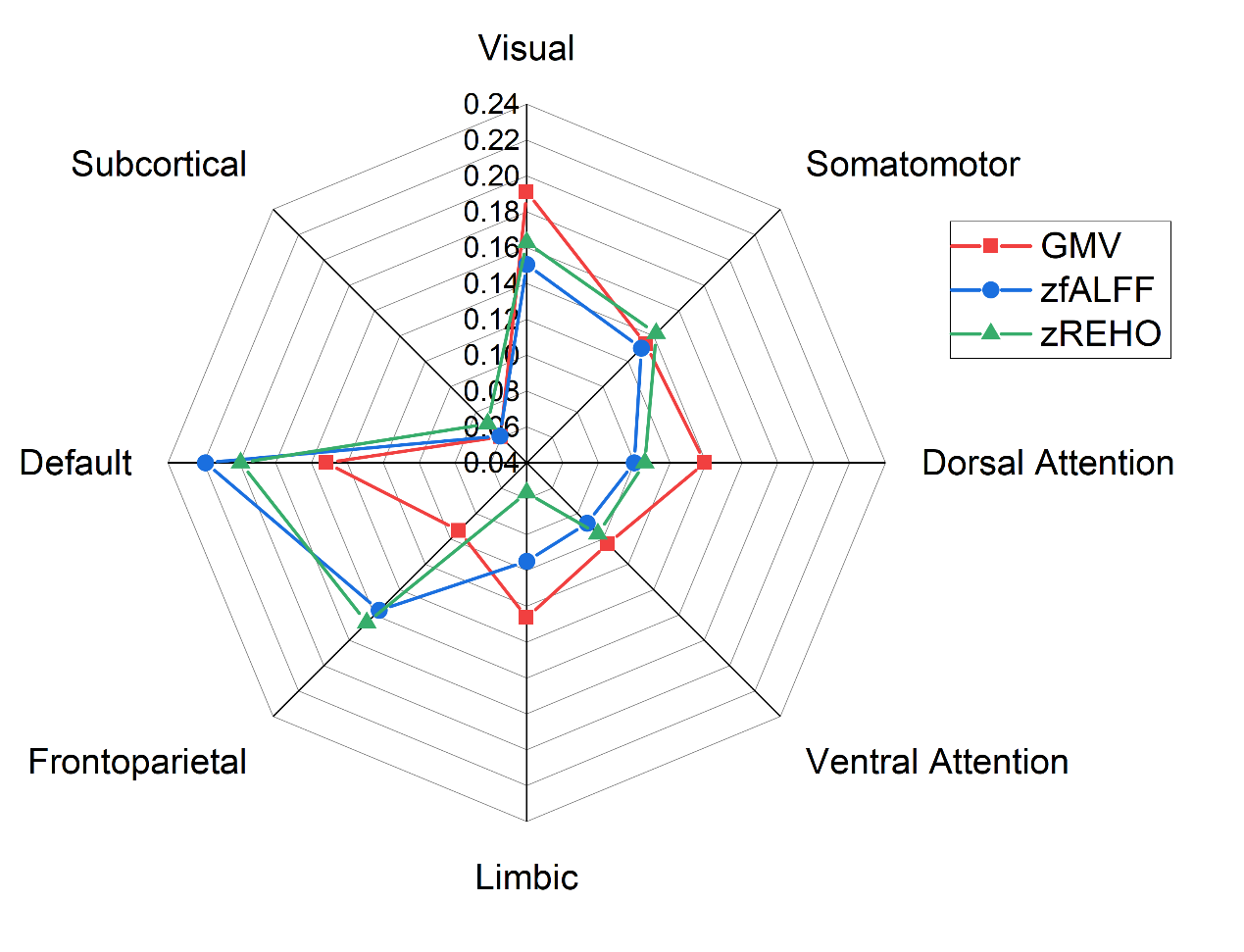


Figure S5. Radar chart for distribution of eight networks module for the combined classifier between DS and NDS groups. The numbers on the radar chart represent the proportion of the network.


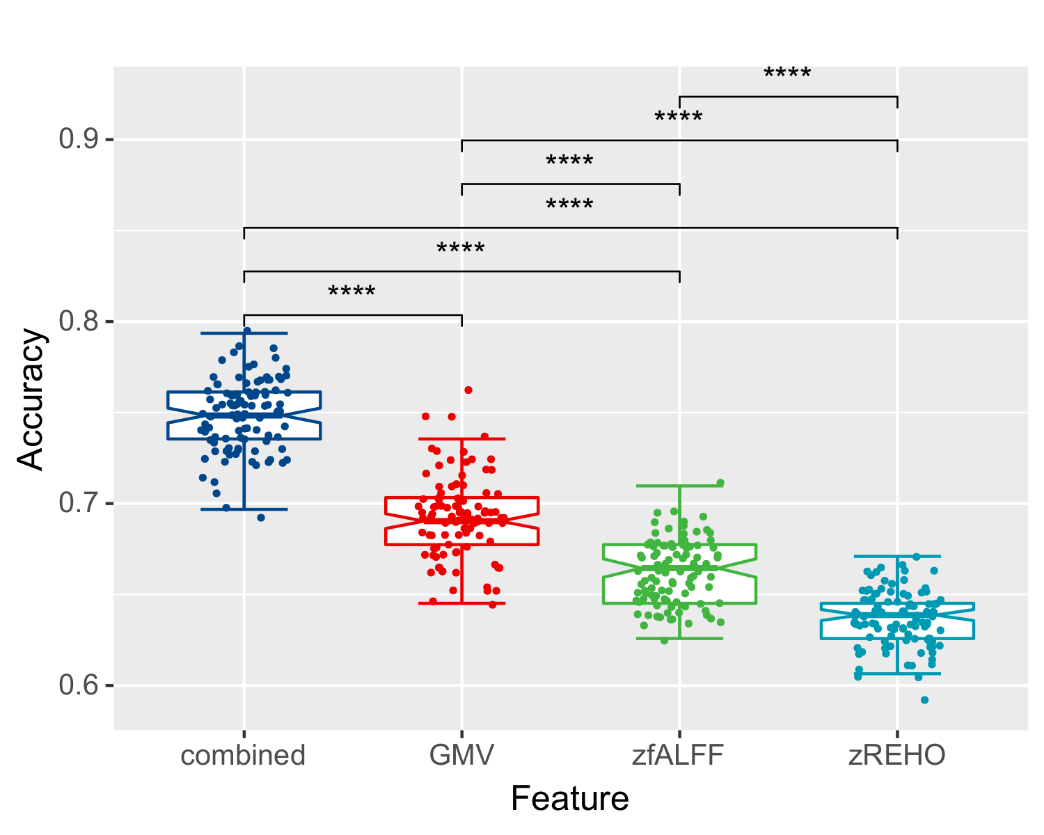


Figure S6. Comparison of accuracy of SVM classification adding duration as a feature between DS and NDS groups (101 times 10-fold cross-validation results).

**References**

1. Wang X, Yao S, Kirkpatrick B, Shi C, Yi J. Psychopathology and neuropsychological impairments in deficit and nondeficit schizophrenia of Chinese origin. *Psychiatry research.* 2008;158(2):195-205.

2. Mueser KT, Curran PJ, McHugo GJ. Factor structure of the Brief Psychiatric Rating Scale in schizophrenia. *Psychological Assessment.* 1997;9(3):196-204.

3. Cohen AS, Saperstein AM, Gold JM, Kirkpatrick B, Carpenter WT, Jr., Buchanan RW. Neuropsychology of the deficit syndrome: new data and meta-analysis of findings to date. *Schizophr Bull.* 2007;33(5):1201-1212.

4. Andreasen NC. Negative symptoms in schizophrenia. Definition and reliability. *Arch Gen Psychiatry.* 1982;39(7):784-788.

5. Blanchard JJ, Cohen AS. The structure of negative symptoms within schizophrenia: implications for assessment. *Schizophr Bull.* 2006;32(2):238-245.

6. Galderisi S, Mucci A, Buchanan RW, Arango C. Negative symptoms of schizophrenia: new developments and unanswered research questions. *The lancet Psychiatry.* 2018;5(8):664-677.

7. Rajapakse JC, Giedd JN, Rapoport JL. Statistical approach to segmentation of single-channel cerebral MR images. *IEEE Trans Med Imaging.* 1997;16(2):176-186.

8. Tohka J, Zijdenbos A, Evans A. Fast and robust parameter estimation for statistical partial volume models in brain MRI. *Neuroimage.* 2004;23(1):84-97.

9. Ashburner J. A fast diffeomorphic image registration algorithm. *NeuroImage.* 2007;38(1):95-113.

10. Krafnick AJ, Flowers DL, Luetje MM, Napoliello EM, Eden GF. An investigation into the origin of anatomical differences in dyslexia. *The Journal of neuroscience : the official journal of the Society for Neuroscience.* 2014;34(3):901-908.

11. Xie Y, Cui Z, Zhang Z, et al. Identification of Amnestic Mild Cognitive Impairment Using Multi-Modal Brain Features: A Combined Structural MRI and Diffusion Tensor Imaging Study. *Journal of Alzheimer's disease : JAD.* 2015;47(2):509-522.

12. Cui Z, Su M, Li L, Shu H, Gong G. Individualized Prediction of Reading Comprehension Ability Using Gray Matter Volume. *Cerebral cortex.* 2018;28(5):1656-1672.

13. Biswal B, Yetkin FZ, Haughton VM, Hyde JS. Functional Connectivity in the Motor Cortex of Resting Human Brain Using Echo-Planar Mri. *Magnet Reson Med.* 1995;34(4):537-541.

14. Zang YF, He Y, Zhu CZ, et al. Altered baseline brain activity in children with ADHD revealed by resting-state functional MRI. *Brain Dev.* 2007;29(2):83-91.

15. Zang Y, Jiang T, Lu Y, He Y, Tian L. Regional homogeneity approach to fMRI data analysis. *Neuroimage.* 2004;22(1):394-400.

16. Yeo BT, Krienen FM, Sepulcre J, et al. The organization of the human cerebral cortex estimated by intrinsic functional connectivity. *Journal of neurophysiology.* 2011;106(3):1125-1165.
